# A Dress Is Not a Yes: Towards an Indirect Mouse-Tracking Measure of Men’s Overreliance on Global Cues in the Context of Sexual Flirting

**DOI:** 10.1007/s10508-023-02798-x

**Published:** 2024-02-07

**Authors:** Ingo Landwehr, Katrin Mundloch, Alexander F. Schmidt

**Affiliations:** 1 Institute of Psychology, Social and Legal Psychology, Johannes Gutenberg University Mainz, Binger Str. 14-16, 55122 Mainz, Germany; 2 Independent Researcher, Nuremberg, Germany

**Keywords:** Sexual arousal, Sexual decision-making, Sex drive, Sexual objectification, Mouse-tracking

## Abstract

Assessing another person’s intention to flirt and, relatedly, their sexual interest is based on the interpretation and weighting of global (e.g., clothing style) and specific (e.g., facial expression) cues. Since cue incongruency increases the risk of erroneous judgments and thus can entail undesirable outcomes for both parties involved, detection of an individual propensity for overly relying on global (sexual) rather than specific (affective) cues is of social and clinical-forensic importance. Using a purpose-designed and pre-validated stimulus set, we developed a mouse-tracking task as an indirect behavioral measure for males’ overreliance on global cues (OGC) in the context of sexual flirting. In a convenience sample of heterosexual cisgender men (*N* = 79), experimentally induced sexual arousal was shown to increase the probability of OGC as a function of task difficulty (i.e., congruent or incongruent combinations of global and specific cues displayed by a potential female flirting partner). While error rate and reaction time proved to be indicators of OGC, the spatial measures maximum deviation and area under the curve provided less consistent results. In addition, error rate suggested sex drive and sexual objectification to act as moderators of the relationship between sexual arousal and OGC. Exploratory analysis further revealed a theoretically meaningful pattern of correlations between mouse-tracking measures and self-report measures of problematic (e.g., disinhibited, exploitative) sexuality. Implications of the results are discussed and a framework for differentiating potential causes of OGC (i.e., misperception, lack of self-control, and egocentric hedonism) is proposed.

**Supplementary Information:**

The online version contains supplementary material available at 10.1007/s10508-023-02798-x.

## Introduction

Sexual interest in a woman sparks a heterosexual man’s desire to approach her (Welsch et al., 2020). Prior to a sexually motivated approach, it is advantageous for the man to assess whether the woman is more likely to reciprocate his sexual interest or not, i.e., whether she engages in flirting with him, as flirting is commonly understood to signal sexual availability (Buss, 1988).^1^ In the initial phase of flirting, such assessment is usually based solely on visually discernible cues, since verbal exchange has not yet taken place. For the purpose of assessment, cues that are specifically directed at the male interpreter of these cues (e.g., facial expression, body language) appear to be particularly promising, in contrast to cues that—although perhaps perceived as sexually appealing—are not specifically directed at him (e.g., physical attractiveness, provocative clothing).

Overreliance on such nonspecific cues obviously increases the risk of misjudging a woman’s (lack of) sexual interest toward the assessing male. As a result, sexually motivated approaches based on such nonspecific cues are more likely to have negative consequences for one or both parties involved, ranging from mild frustration to norm-violating (e.g., sexually offensive) behavior, depending on whether (and when) the man eventually recognizes the diverging interests and how he subsequently responds. Individual differences in the interpretation and weighting of cues in the context of flirting thus prove significant not only from a merely social but also from a clinical-forensic perspective, the latter especially in light of research showing that a subgroup of men particularly prone to perceive sexual intent in women is also more likely to engage in sexual coercion (Farris et al., 2008).

### Global vs. Specific Flirting Cues

In the broad context of social encounters, nonverbal cues can be divided into cues that are (1) global, i.e., situationally stable (e.g., physical attractiveness, clothing style) and (2) specific, i.e., situationally variable (e.g., body language, facial expression). Global and specific cues differ in what information they carry for a particular (dyadic) social interaction, since specific cues by their nature pertain to that interaction only, whereas global cues do not, but at most refer to a general intention or mood. Failure to adequately account for this difference in informational value in the context of flirting increases the likelihood of overreliance on global cues (OGC), which means that inadequate (i.e., nonspecific) information is used to assess another person’s current (lack of) sexual interest. For example, while in most public social contexts (e.g., when going for a night out) it would be appropriate to think of a woman’s clothing as independent of her attitude toward a particular person, her facial expression should be understood as a cue directed specifically at the person with whom she is having eye contact at that very moment (Adams & Kleck, 2005).

Since facial expressions reliably distinguish between different situations and interaction partners, primary attention should be paid to them when assessing another person’s intention during a social interaction. Especially if no verbal communication has taken place yet, a facial expression conveying positive affect can be understood as a possible invitation to further contact and approach (Miles, 2009). Using a mouse-tracking (MT) paradigm, Treat et al. (2020) showed that men at greater risk of exhibiting sexual aggression against women are more inclined to evaluate women’s sexual interest based on inappropriate global rather than appropriate specific cues, i.e., based rather on normative attractiveness and provocative clothing than on the affect a woman displays. The correlations found by Treat et al. (2020) point to the also theoretically plausible link between individual propensity for OGC and an increased risk for problematic sexual behavior.

### Sexual Arousal and Cue Relevance

Motivational (visceral) states affect decision-making and selection behavior in favor of need-congruent stimuli, i.e., stimuli that match the state of deprivation an individual is currently experiencing (e.g., food when feeling hungry; Seibt et al., 2006). As has been shown, this is also true for sexual arousal (Ariely & Loewenstein, 2006; Skakoon-Sparling et al., 2015), as sexual arousal increases the appeal of sexual stimuli by the prospect of sexual pleasure (Meston & Buss, 2007). As a result, sexually aroused individuals—especially men—are more prone to engage in sexually disinhibited behavior (Imhoff & Schmidt, 2014; Wiemer et al., 2023). This also applies to situations in which inhibition of sexual behavior would be socially appropriate (e.g., when sexual interest is not reciprocated), while acting out is sanctioned (Bouffard, 2011). In light of this, it seems obvious to investigate whether the “motivational myopia” (Ditto et al., 2006) created by sexual arousal “in the heat of the moment” (Ariely & Loewenstein, 2006) affects men’s cue-based assessment of potential flirting partners, i.e., to what extent sexually aroused men tend to disregard a discrepancy between their own and a woman’s sexual interest by focusing on global sexual rather than specific affective cues. Such an approach is further warranted by findings suggesting that a state of sexual arousal in men might prominently contribute to misunderstandings in sexual communication (Rerick et al., 2019).

### Mouse-Tracking Selection Tasks

The premise underlying MT is that motor movements contain information about task-relevant cognitive processes occurring within the same time frame (Spivey & Dale, 2006). To access this information, MT continuously captures the mouse cursor movements toward a response option caused by participants’ hand movements during a computer experiment (Freeman & Ambady, 2010a). Specifically, MT paradigms have been repeatedly used to measure the temporal unfolding of decision-based selection processes by asking participants either to choose between two competing stimuli (e.g., healthy vs. unhealthy food; Stillman et al., 2017) or to assign a single stimulus to one of two competing categories (e.g., feminine vs. masculine stereotypes; Freeman & Ambady, 2009). In both designs, participants are required to select one of two options and are thus subject to a (more or less pronounced) selection conflict.

Measures calculated by MT software (Freeman & Ambady, 2010b) are reaction time (RT), maximum deviation (MD), area under the curve (AUC), and error rate (ER). RT corresponds to the time participants need to move the mouse cursor from the starting field to the target. As has been frequently shown, response latency increases as a function of task difficulty (e.g., Hick, 1952). Consequently, a stronger conflict in the course of a selection task should be reflected in the time it takes participants to decide on one of the offered options. MD and AUC represent the spatial path of a mouse trajectory in terms of numerical parameters. While MD is defined as the point of an empirical trajectory farthest from an ideal straight line between the starting field and the target, AUC refers to the area between the ideal straight line and the empirical trajectory. High values for these two measures thus reflect an increased curvature of the mouse trajectory and point to a correspondingly high attraction of the non-selected option (thereby indicating a temptation or distraction of some kind emanating from this option). A normative error occurs when an option is not only partially headed for, but actually chosen, even though this choice contradicts a given instruction (e.g., when ice cream is chosen instead of broccoli, even though the instruction calls for making the choice in accordance with a healthy diet).

Smith et al. (2018) used an MT paradigm to examine the real-time dynamics of men’s decisions about women’s sexual interest by presenting full-body images of women to be assigned to one of two categories (“sexually interested” or “rejecting”). The depicted women varied in affect (sexual interest or rejection, i.e., a specific cue), clothing style (provocative or conservative, i.e., a global cue), and normative attractiveness (attractive or unattractive, i.e., another global cue). The authors’ conjecture was that normatively incongruent cue combinations would lead to increased selection difficulty. While overall participants’ assessments were normatively correct (i.e., consistent with the specific cue), for both global cues the expected congruency effect was found, as more errors occurred when specific and global cues were presented incongruently (e.g., when the woman showed rejection but at the same time wore provocative clothing). In addition to assessment accuracy, Smith et al. (2018) tracked mouse movements to examine how easy or difficult it was for participants to choose between the two categorical options. As expected, the mouse trajectories in incongruent trials were found to converge more closely to the normatively incorrect option than those in congruent trials, suggesting increased selection difficulty. Thus, a congruency effect was found not only for the selection outcome in terms of accuracy, but also for the selection process manifested in mouse trajectories.

### Sex Drive and Sexual Objectification as Potential Moderators

*Sex drive* refers to the strength of dispositional sexual motivation (Baumeister et al., 2001). Individual differences in sex drive are evident in the frequency of sexual desire, the variety of (desired or performed) sexual acts, the number of (desired or real) sexual partners, the frequency of sexual fantasies and masturbation, and the willingness to make sacrifices in other areas of life for the sake of sex (Baumeister et al., 2001). Since Moholy et al. (2015) demonstrated that individuals with high sex drive are less successful in controlling their impulse-driven behaviors in a state of sexual arousal, it seems justified to speculate that such individuals may also be more prone to overly rely on global sexual cues, i.e., cues that are congruent with their sexual desire.

Sexual objectification is a form of perception or action in which a person is regarded or used as a mere object of sexual desire or as a sexual resource by reducing them to their body or to specific sexual functions of their body (Bartky, 1990). Sexual objectification also makes the sexually desired person appear more attainable by denying them relevant needs and interests of their own (e.g., Vaes et al., 2011), especially since these needs and interests may constitute an obstacle to the objectifier’s intentions. Accordingly, if sexual objectification leads to the subjective perception that one’s own sexual interest is not countered by an opposing interest, or that such an opposing interest is at least irrelevant, OGC should become more likely, as in this case, there is no motivation to pay attention to cues that might indicate such an opposing interest (i.e., specific affective cues). As has been demonstrated, sexual objectification can be provoked or enhanced by presenting images of women in sexualized clothing (e.g., Gurung & Chrouser, 2007). In the current study, a woman wearing a sexually appealing outfit was shown as one of two stimuli in each trial. This choice of visual stimuli is also in line with research findings that an objectifying gaze (the “male gaze”; Gervais et al., 2013) is the most common expression of sexual objectification in everyday life (Holland et al., 2016).

### Current Study

Previous studies were successful in using MT to reveal individual difficulties in solving selection tasks (e.g., Lim et al., 2018; Smith et al., 2018). Thus, it seemed reasonable to assume that an MT selection task would also be a suitable method for measuring individual propensity for OGC in a state of sexual arousal, provided that (non)compliance with an instruction given to solve the MT task would be reflected in behavioral correlates of OGC (i.e., in the four MT measures RT, MD, AUC, and ER). Normative errors in particular as well as an altered RT, but also more curved mouse trajectories pointing to selection difficulties, could then be interpreted as indicators of an increased individual risk for real-life occurrence of OGC and its possible negative consequences. Such a conclusion would be even more warranted if (1) constructs of socially inappropriate or dysfunctional (e.g., disinhibited, exploitative) sexuality could be found that moderate the relationship between sexual arousal and OGC as indicated by MT and if (2) indicators of OGC were positively correlated with self-reports of problematic sexual attitudes, behaviors, experiences, and motivations (i.e., if they shared a nomological network with traits related to problematic sexuality).^2^

Building on the findings mentioned above, we extended earlier approaches (e.g., Smith et al., 2018; Treat et al., 2020) by (1) designing two different trial types, with each trial type requiring a choice between two competing stimuli depicting the same woman displaying different cue combinations (see Stimuli and Trial Types section for details) and by (2) introducing repeated measurements (about 20 min apart). We expected the incongruent trials to cause more pronounced selection difficulties than the congruent trials, i.e., that a congruency effect would occur for both the selection outcome and the selection process. In accordance with their presumed different potential to elicit selection conflicts, we referred to the incongruent trial condition as the high conflict (HC) condition and the congruent trial condition as the low conflict (LC) condition. Additionally, sexual arousal was induced between T1 and T2, such that the baseline condition at T1 was completed in a “cold” visceral state, whereas the putative effect of “hot” states on decision-making (Ariely & Loewenstein, 2006; Skakoon-Sparling et al., 2015), i.e., on the selection of potential flirting partners, could be observed at T2 and relative to T1. While we expected selection in line with the given instruction to be generally more difficult in a state of sexual arousal at T2 than in the baseline condition at T1, we also expected this difficulty to increase further when, in a state of sexual arousal, the given instruction would conflict with a spontanous preference for need-congruent (global) sexual stimuli, i.e., when the incongruent cue combinations displayed in HC trials would interact with a state of sexual arousal.

As potential moderators of the hypothesized relationship between sexual arousal and OGC, we chose two constructs based on both theoretical considerations and previous research (i.e., sex drive and sexual objectification). Furthermore, to test our conjecture of a nomological network of OGC and aspects of problematic sexuality, we had several standard self-report questionnaires answered and correlated their scale scores with the MT measures, expecting a theoretically meaningful pattern of correlations. In addition to these established questionnaires, we had participants answer a self-constructed questionnaire on their propensity for socially problematic (e.g., manipulative) flirting behavior (see Measures Section for details on all questionnaires). We made this particular addition since we also chose a flirting framework for our selection task to draw on an everyday real-world experience often accompanied by a sexual objective (Henningsen, 2004; Moore, 2010). Moreover, flirting usually takes place dyadically, so prioritizing specific affective over global sexual cues should provide a favorable (i.e., socially appropriate as well as more promising) basis for selection. The instruction for the flirting task (i.e., “Click on the woman who is more likely to flirt with you right now”) was designed to focus on the perceived sexual interest (present or absent) of the depicted women, whereas participants’ own sexual interest was not addressed and should therefore be irrelevant to the decision.

#### Hypotheses

The above research findings and the considerations derived from them led us to the following hypotheses:

1. HC trials will differ from LC trials by (1) increased RT, (2) increased MD, (3) increased AUC, and (4) increased ER (i.e., main effect of trial type).
2. Sexual arousal at T2 will result in (1) increased RT, (2) increased MD, (3) increased AUC, and (4) increased ER compared to T1 (i.e., main effect of time).
3. The influence of sexual arousal as formulated in Hypothesis 2 will be more evident in HC trials than in LC trials (i.e., interaction of trial type and time).
4. Higher sex drive will be associated with a stronger manifestation of the interactional relationships as formulated in Hypothesis 3.
5. A higher propensity for sexual objectification will be associated with a stronger manifestation of the interactional relationships as formulated in Hypothesis 3.

## Method

### Participants

Initially, the convenience sample comprised 89 adult cisgender males recruited through flyers, posters, and email distribution lists within a medium-sized German college town, including recruitment on campus and at local soccer clubs. Participants were required to self-identify as exclusively or predominantly heterosexual, thereby indicating a preference for women as potential sexual partners. Nine participants had to be excluded from further analyses after evaluating their sexual orientation and manipulation checks. Of these, three reported values > 2 on the Kinsey scale (Kinsey et al., 1948), so that for them the required sexual attraction of the female stimuli was not ensured. Seven participants stated that they had not been sexually aroused at all by the erotic audio story used to induce sexual arousal between T1 and T2. One of the nine excluded participants met both exclusion criteria. All remaining participants in the effective sample (*N* = 79) stated that they had answered the questionnaires honestly.^3^

The majority (70%) of the effective sample consisted of university students from various disciplines. Participants’ age ranged from 18 to 50 years (*M* = 25.47, *SD* = 5.62). Most participants (68%) classified themselves as exclusively heterosexual (level 0 on the Kinsey scale), while the rest stated that occasionally they also had homosexual fantasies or contacts (24% level 1, 8% level 2). About half (51%) were in a committed relationship. The highest educational attainment reported by 44% was a university degree, whereas 51% reported the general higher education entrance qualification (“Abitur”) and 5% the secondary school leaving certificate (“Mittlerer Schulabschluss”). Participants either had normal vision or wore visual aids. All were right-handed or could easily operate a mouse with their right hand. As an incentive, participants could choose between € 5 (about US$ 5.5; chosen 39 times) and course credit (chosen 40 times).

### Stimuli and Trial Types

Prior to the present study, 378 pictorial stimuli (i.e., full-body photographs of adult women in both casual and rather revealing clothing with different facial expressions) were created and validated (for detailed information on the full stimulus set, see Landwehr & Mundloch, 2023). For the MT paradigm introduced here, a subset of these stimuli consisting of 192 images was used and divided into two parallel sets (each stimulus was presented only once). These parallel sets were matched on mean and distributed sexual attractiveness values (for more details on the stimuli used in this study see the Electronic Supplement section ES1). The pictorial stimuli had to meet certain criteria such as (1) high authenticity in terms of both individually chosen clothing (models brought their own clothes which they would actually wear in situations similar to the task’s conditions, namely either when “visiting a good friend in casual clothing to hang out together” or “having a night out in sexy clothing with the general intention to flirt”) and affective facial expressions matching two to-be-imagined social situations (in which models should either flirt with a person they are sexually interested in or unequivocally reject an obnoxious person as a flirting partner), (2) a clear contrasting effect per model between the two outfits (Casual vs. Sexy) as well as the two facial expressions (Flirting vs. Rejecting), and (3) standardization of various external factors, such as lighting and final image editing. Since Van den Stock et al. (2007) demonstrated that body language greatly influences the perception of facially communicated emotion, we (4) asked our models to maintain a standardized neutral posture regardless of their respective facial expression. In this way, we ensured that potential effects could be clearly attributed to variation in global (clothing) and specific (facial expression) cue combinations.

In contrast to Smith et al. (2018), we used an MT paradigm in which participants did not assign single stimuli to one of two categories but had to select one of two stimuli presented simultaneously, i.e., two images of the same woman. Importantly, this design allowed us to avoid a confounding effect of differences in participants’ subjective perceptions of different women’s attractiveness. Also, because the differences between the two images were entirely due to the clothing and facial expression cues displayed by the same woman, we expected the selection task to be generally quite easy to solve, emphasizing the diagnostic relevance of any instruction-inconsistent behaviors (i.e., indicators of OGC) that might manifest in the four MT measures.

In the HC condition, one of the images showed a woman in casual everyday clothing with a flirtatious facial expression (the Casual × Flirting combination), while the other image showed her in rather sexy clothing with a rejecting facial expression (the Sexy × Rejecting combination). This juxtaposition was based on the assumption that both a flirtatious facial expression and a sexually suggestive outfit are cues that men perceive as (sexually) appealing and thus facilitate male sexual selection (e.g., Grammer et al., 2004; Walsh & Hewitt, 1985). At the same time, it was reasonable to assume that both a rejecting facial expression and inconspicuous clothing would not be perceived as equally positive. Instead, these cues should be perceived as rather neutral (in the case of the casual outfit) or even as negative (in the case of the rejecting facial expression), which is why male sexual selection should not be facilitated by them and might even be inhibited (e.g., Goodboy & Brann, 2010; Moore, 1998). Since in the HC condition both images were incongruent, i.e., both contained a positive and a non-positive cue, a prompt to choose between them could be expected to evoke a selection conflict of some degree.

**Fig. 1:**
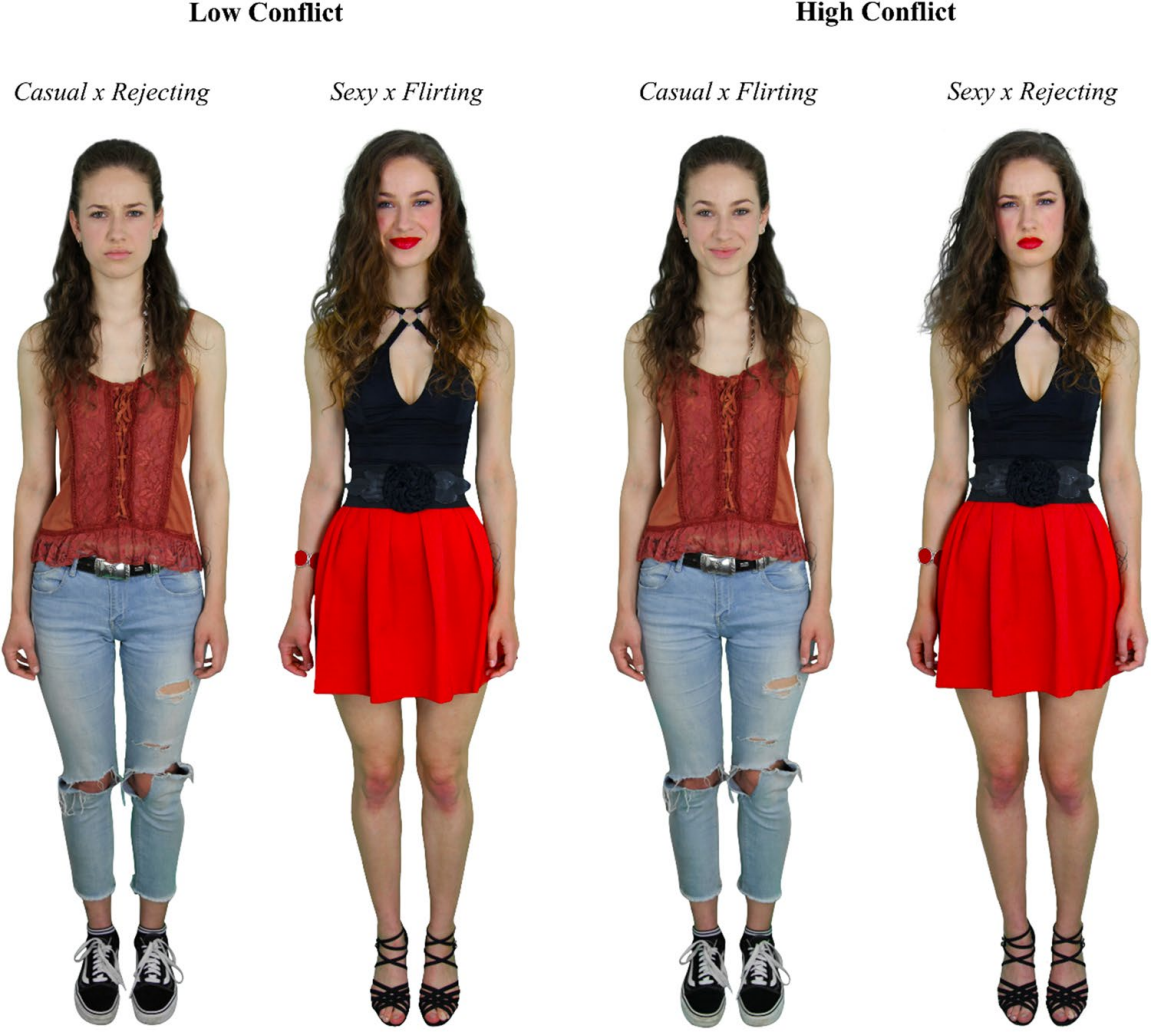
Trial types and cue combinations

We intermixed the HC trials with trials of the LC condition. In the LC condition, one of the images showed a woman with both positive cues (the Sexy × Flirting combination), while the other image showed her with both non-positive cues (the Casual × Rejecting combination). When confronted with two such juxtaposed congruent images, the participants’ choice was expected to fall clearly on the image showing the Sexy × Flirting combination, as this combination would correspond to both the more sexually appealing (clothing) option and the affective (facial expression) option normatively consistent with the given instruction to select “the woman who is more likely to flirt with you right now.” Fig. 1 shows the HC and LC conditions along with their respective cue combinations.

Using Krippendorff’s α coefficient (Hayes & Krippendorff, 2007), interrater reliability for the overall discriminability of the two clothing cues, Casual and Sexy, as well as the two facial expression cues, Flirting and Rejecting, was determined. While reliability was high for the clothing cues (*r*_α_ = .82) and very high for the facial expression cues (*r*_α_ = .93), most importantly, for all images—which were presented in the same pairwise combinations as they would later form the HC and LC trials of the experiment—the cues contained were assigned to the respective normatively correct category (i.e., Casual or Sexy, Flirting or Rejecting) by the majority of raters. Also, as expected, images of the Flirting condition were rated as more sexually attractive than images of the Rejecting condition (*p* < .001, *d*_z_ = 1.32), while images of the Sexy condition were rated as more sexually attractive than images of the Casual condition (*p* < .001, *d*_z_ = 0.94). Crucially, the expected group difference in perceived sexual attractiveness was particularly pronounced between the two LC cue combinations (1) Sexy × Flirting and (2) Casual × Rejecting (*p* < .001, *d*_z_ = 2.04), while, as anticipated, no group difference emerged between the two HC cue combinations (1) Casual × Flirting and (2) Sexy × Rejecting (*p* = .197, *d*_z_ = 0.12). These pretest results clearly corroborated the paradigm’s fundamental premises in terms of either distinct or analogous attractiveness-related stimulus effects (see ES1 for detailed information).

### Measures

We employed seven self-report questionnaires assessing problematic sexuality, presented sequentially in a fixed order to maximize between-subjects effects. To avoid participant fatigue, shortened versions were used if available. Acceptance of rape myths was assessed using the Acceptance of Modern Myths about Sexual Aggression Scale (AMMSA; Gerger et al., 2007; German short version Süssenbach & Bohner, 2011; nine five-point items from 1 = *does not apply at all* to 5 = *totally applies*). Rape myths are descriptive or prescriptive beliefs about rape (i.e., about its causes, context, consequences, perpetrators, victims, and their interactions) that serve to deny, trivialize, or justify men’s sexual violence against women (Gerger et al., 2007). The Hypersexual Behavior Inventory-19 (HBI-19; Reid et al., 2011; German version Klein et al., 2013; eight five-point items from 1 = *does not apply at all* to 5 = *totally applies*) assesses hypersexual urges, thoughts, fantasies, and behavior associated with clinically relevant distress or functional impairment in important life domains. The Sexual Desire Inventory-2 (SDI-2; Spector et al., 1996; German short version Kuhn et al., 2014; ten eight-point items, two items from 1 = *never* to 8 = *more than once a day*, five items from 1 = *no desire* to 8 = *strong desire*, two items from 1 = *much less desire* to 8 = *much more desire*, one item from 1 = *unimportant* to 8 = *extremely important*) measures sexual desire based on two subscales (Dyadic Sexual Desire and Solitary Sexual Desire) that can be combined into a total score. To assess individual differences in sexual inhibition and excitation, we used the Sexual Inhibition/Sexual Excitation Scales (SIS/SES; Janssen et al., 2002; German short version Turner et al., 2014; SIS: eight five-point items from 1 = *does not apply at all* to 5 = *totally applies*; SES: six five-point items from 1 = *does not apply at all* to 5 = *totally applies*). The revised Sociosexual Orientation Inventory (SOI-R; Penke & Asendorpf, 2008; nine five-point items, three items from 1 = *0* to 5 = *8 or more*, three items from 1 = *I do not agree at all* to 5 = *I agree completely*, three items from 1 = *never* to 5 = *almost every day*) measures individual preferences for impersonal sex and casual sexual encounters. Since SDI-2, SES, and SOI-R represent different (yet usually highly intercorrelated) facets of sex drive, an aggregated index of their *z*-standardized values was calculated to measure sex drive on a higher level of aggregation in the present study. The Sexual Objectification of Others Inventory (SOOI; Anslinger, 2019; ten five-point items from 1 = *strongly disagree* to 5 = *strongly agree*) was used to assess the propensity to perceive women primarily in terms of their sexual appeal and functionality based on two factors (Instrumental Objectification and Visual Objectification).^4^ Participants also answered the Socially Undesirable Sexual Selection Scale (SUSS; Schmidt, 2023; 13 items to be answered on a visual analogue scale from 0 = *very unlikely* to 100 = *most likely*). This unidimensional scale was developed to capture indications of manipulative flirting behavior. We assumed that willingness to use manipulative flirting tactics in particular would be associated with an increased likelihood of OGC, as both phenomena stem from an egocentric selection criterion, i.e., the sexual interest of the selecting male, as opposed to an assessment of the woman’s interest (example item: “I am willing to deceive the woman so that she will have sex with me”; see ES2 for validity details).

Following T2, participants answered three manipulation check questions concerning (1) the perceived extent of sexual arousal evoked by the erotic audio story (i.e., Arousal_Story_; “How sexually arousing did you find the story you heard?” from 1 = *not arousing at all* to 5 = *very arousing*), (2) the perceived extent of current sexual arousal (i.e., Arousal_Now_; “How sexually aroused are you right now?” from 1 = *not aroused at all* to 5 = *very aroused*), and (3) their honesty in answering the questionnaires (“How honestly have you answered the questions during this study?” from 1 = *not honest at all* to 5 = *completely honest*).

For the MT task, RT, MD, AUC, and ER aggregated per trial type and measuring time were used as dependent variables. An initial preparation of these data was carried out with the software MouseTracker (Freeman & Ambady, 2010b). Each recorded trajectory was normalized and divided into 101 time steps.

### Apparatus and Procedure

The experiment was conducted using a custom laptop with a 17-inch screen set to a resolution of 1366 × 768 pixels, standard headphones, and a standard optical mouse with a resolution of 800 DPI connected via USB cable. The software MouseTracker (Freeman & Ambady, 2010b) was used to record RT, MD, AUC, and ER. The software Inquisit 5 Lab (Millisecond, 2016) was used for presenting the questionnaires and recording participants’ answers.

First, participants provided consent to take part in a study on flirting behavior. They were informed that they would be listening to an erotic audio story and answering various questions about their sexuality. They were also assured that all data would be collected anonymously, and that they could withdraw from the study at any time without any disadvantages. They then read the description of the MT task which explained that they should move the mouse as fast as possible while answering as correctly as possible according to the given instruction (i.e., “Click on the woman who is more likely to flirt with you right now”). T1 started with four practice trials (unbeknownst to the participants and excluded from the analyses) after which 48 trials followed in a fixed-randomized order to maximize between-subjects effects. Of these trials, 24 belonged to the HC condition and 24 to the LC condition. Following T1, participants completed seven questionnaires (see Measures section for details). Next, they were asked to put on headphones and listen to a 332 s audio story of explicit erotic content used to induce sexual arousal (taken from Imhoff & Schmidt, 2014, Study 1). Subsequently, T2 followed with two practice trials and, again, 48 regular trials. Finally, participants answered three manipulation check items on state sexual arousal and honesty (see Measures section for details). The extent of sexual arousal evoked by the erotic audio story was assessed only after T2 and not immediately after the audio story (1) to prevent an interruption/a distraction between the induction of sexual arousal and the onset of T2 by a question whose answer requires cognitive processing and thus could diminish the extent of sexual arousal and (2) to avoid overly emphasizing the significance of sexual arousal for the subsequent task, so as to preempt altered (e.g., inhibited) response behavior.

**Fig. 2:**
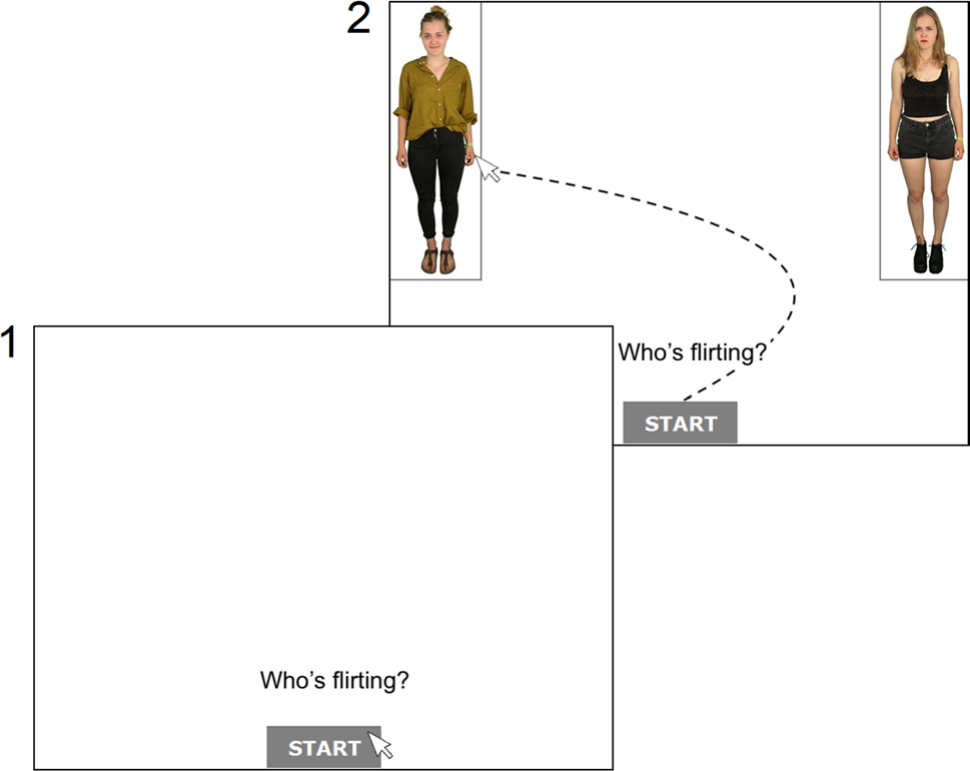
Structure and course of a mouse-tracking trial in the high conflict condition

At the beginning of each MT trial, participants had to click the start button, only after which the two images appeared in the upper left and right corners of the screen (see Fig. 2). Now, participants had to move the mouse within a 1000 ms response window, creating a dynamic starting condition which proved to be methodologically advantageous by enhancing the transfer of decisional processing into mouse movements (Scherbaum & Kieslich, 2017). Participants next had to choose one of the two presented images within a 5000 ms response window. Both response windows were chosen rather liberally (Hehman et al., 2014). If the start button or an image had not been clicked within the respective response window, a prompt in red letters appeared reminding participants to move the mouse faster. A shortened version of the instruction (i.e., “Who’s flirting?”) was displayed above the start button before and during each trial to remind participants of the task at hand.

### Statistical Analyses

Trials of both conditions in which participants took too long to initially move the mouse (> 1000 ms) or to choose one of the two images (> 5000 ms) were discarded from the analyses (0.7% of all trials). Prior to conducting the 2 (Trial Type: HC vs. LC) × 2 (Time: T1 vs. T2) repeated measures ANOVAs, the data were screened for outliers. One data set proved to be a clear outlier according to the Tukey criterion (> 3 interquartile ranges) and was thus excluded from further analyses. The significance level for all calculations was set to 5%. Depending on the statistical method, *R*^2^, η_p_^2^, *d*_z_, and ß are reported as effect sizes.

For the moderation and correlation analyses conducted in this study, MT measures were first converted into trial condition-based difference scores for T1 and T2, respectively (e.g., ΔRT_T1_ = RT_T1_HC_ − RT_T1_LC_). While fluctuations in RT, erratic deviations in mouse movements, and unsystematic errors should occur in both trial conditions, systematic effects were expected to occur only due to the more demanding selection task in the HC condition. These difference scores therefore represent adjusted measures of selection difficulty solely attributable to the incongruent presentation of global and specific cues, and can thus be interpreted as indicative of individual differences in OGC. The presumed moderating effect of (1) sex drive and (2) sexual objectification on the relationship between sexual arousal and OGC was tested by moderated hierarchical regression analyses for each dependent variable with (1) sex drive (in form of the aggregated index) and (2) sexual objectification (in form of the SOOI score) as moderators. Two corresponding MT difference scores from T1 and T2 were used as predictor and criterion for each regression (e.g., ΔMD_T1_ as predictor and ΔMD_T2_ as criterion).

## Results

### Mean Differences

First, in order to test the internal validity of the MT paradigm, a 2 (Trial Type: HC vs. LC) × 2 (Time: T1 vs. T2) repeated measures ANOVA was conducted for each of the MT measures. Crucially, Table 1 indicates a significant main effect of trial type on the four MT measures. As expected, all means were higher in the HC condition than in the LC condition. In the HC condition, participants thus took longer to select an option, were more prone to normative errors, and their mouse trajectories showed greater curvatures than in the LC condition, supporting Hypotheses 1a–d.

**Table 1:** Repeated measures ANOVAs for mouse-tracking measures

Source	RT			MD			AUC			ER		
	*F*(1, 78)	*p*	η_p_^2^	*F*(1, 78)	*p*	η_p_^2^	*F*(1, 78)	*p*	η_p_^2^	*F*(1, 78)	*p*	η_p_^2^
Trial Type	32.10	< .001	.29	36.53	< .001	.32	40.10	< .001	.34	23.55	< .001	.23
Time	28.81	< .001	.27	< 1	.970	< .01	< 1	.410	.01	12.05	.001	.13
Trial Type × Time	4.00	.049	.05	1.78	.187	.02	1.62	.207	.02	9.90	.002	.11

	T1 *M* (*SD*)	T2 *M* (*SD*)		T1 *M* (*SD*)	T2 *M* (*SD*)		T1 *M* (*SD*)	T2 *M* (*SD*)		T1 *M* (*SD*)	T2 *M* (*SD*)	
LC	1669 (383)	1539 (417)		.34 (.24)	.36 (.28)		.62 (.50)	.68 (.64)		.02 (.04)	.02 (.03)	
HC	1753 (377)	1587 (421)		.43 (.26)	.42 (.26)		.84 (.60)	.84 (.67)		.06 (.09)	.10 (.15)	

*N* = 79. AUC = area under the curve; ER = error rate; HC = high conflict condition; LC = low conflict condition; MD = maximum deviation; RT = reaction time

A significant main effect of time (Table 1) emerged for RT and ER, but not for MD and AUC, so that hypotheses 2b and 2c had to be rejected. A comparison of the means further revealed that RT unexpectedly decreased from T1 to T2, which is why Hypothesis 2a had to be rejected as well. However, in line with Hypothesis 2d, ER increased significantly from T1 to T2.

**Fig. 3:**
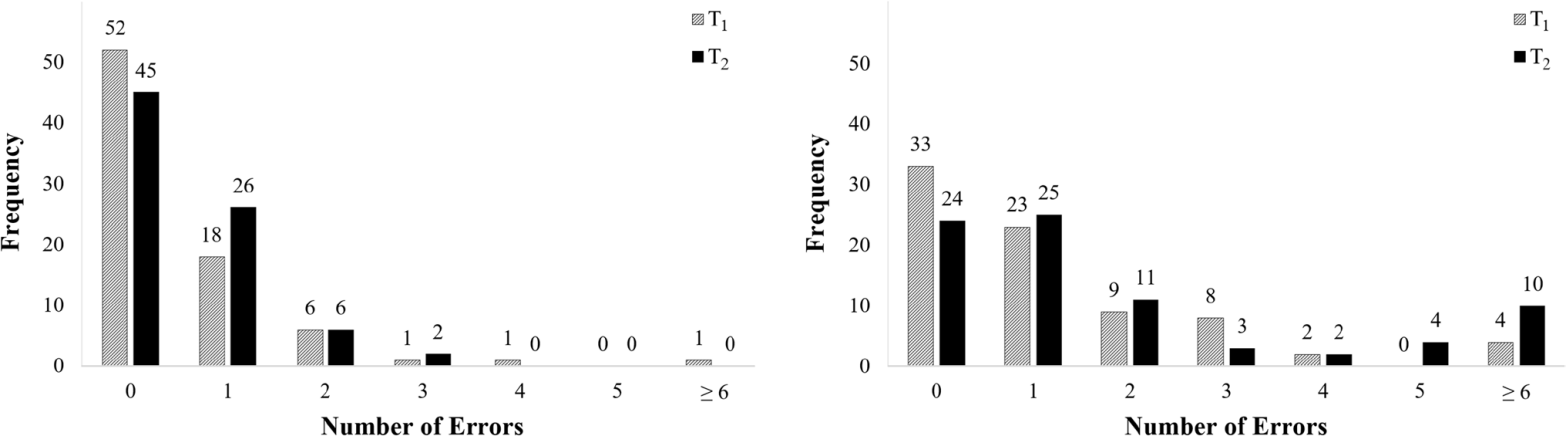
Numbers of errors for both trial conditions. *Notes*. Left chart: low conflict trial condition; right chart: high conflict trial condition

Interaction of trial type and time (Table 1) revealed no significant effect for the spatial MT measures MD and AUC, so Hypotheses 3b and 3c were rejected. Moreover, although an interaction was observed for RT, this effect was not in line with the direction of Hypothesis 3a, as instead of an increase, RT decreased from T1 to T2, even more so in the HC (*p* < .001, *d*_z_ =  − 0.42) than in the LC (*p* < .001, *d*_z_ =  − 0.32) condition. In accordance with Hypothesis 3d, however, an interaction was found for ER: While in the LC condition participants made an equal number of errors at T1 and T2 (*p* = .964, *d*_z_ = 0.01), in the HC condition ER increased significantly from T1 to T2 (*p* < .001, *d*_z_ = 0.46). Error frequencies as a function of trial type and time are shown in Fig. 3.

### Sex Drive and Sexual Objectification as Moderators

**Fig. 4:**
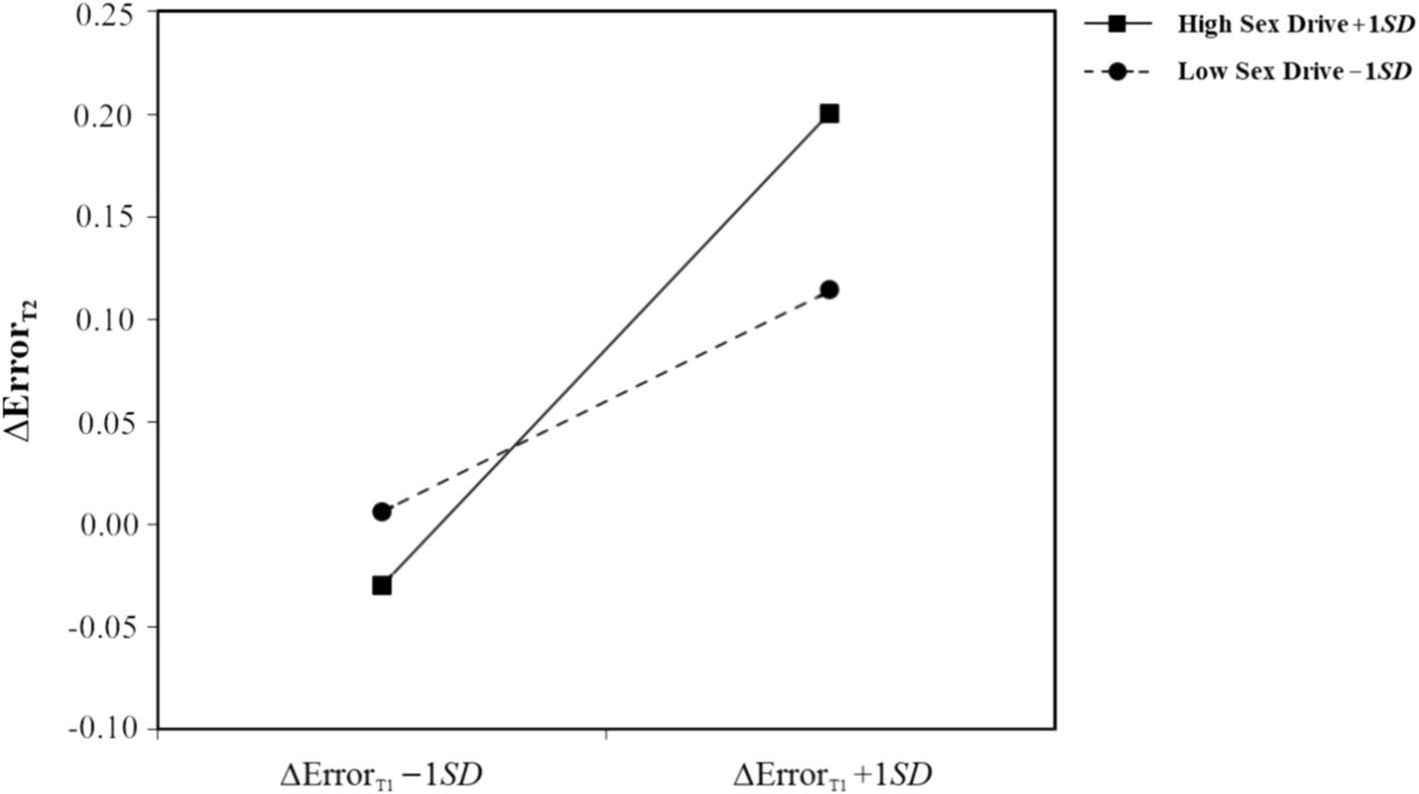
Moderation of sex drive on the relationship between sexual arousal and overreliance on global cues as indicated by error rates

**Fig. 5:**
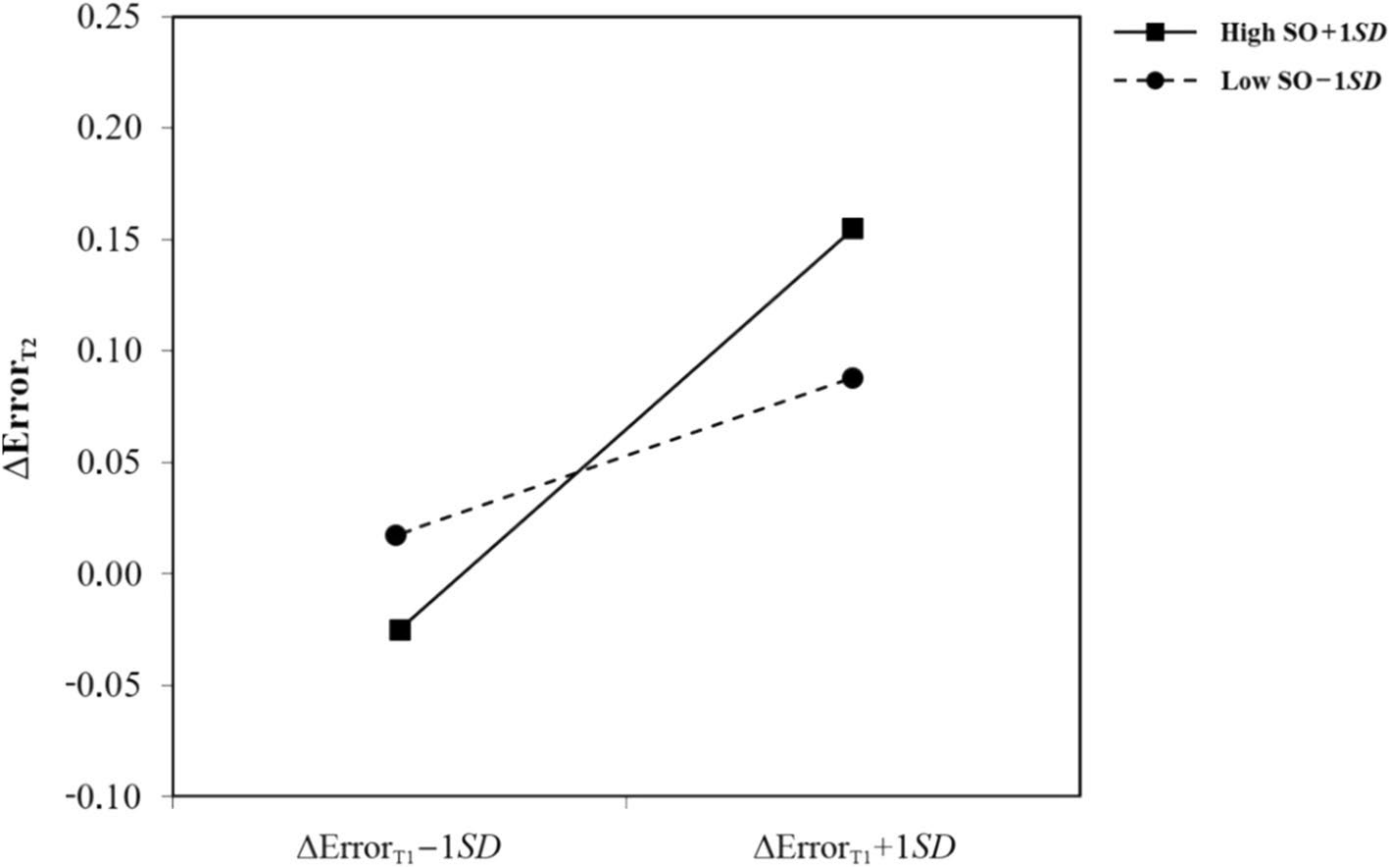
Moderation of sexual objectification on the relationship between sexual arousal and overreliance on global cues as indicated by error rates. *Note*. SO = sexual objectification

Since the mean differences reported above provided preliminary support for the validity of the MT paradigm, as a next step, we conducted moderated hierarchical regression analyses to investigate two potential moderators of the relationship between sexual arousal and OGC. MT difference scores were entered in separate hierarchical regression analyses with (1) sex drive and (2) sexual objectification as moderators. As it turned out, the relationship between sexual arousal and OGC was significantly moderated by both sex drive and sexual objectification only when OGC was operationalized by ER, but not when operationalized by RT, MD, or AUC (see Table 2). Thus, while Hypotheses 4a–c had to be rejected, Hypothesis 4d was supported since in a state of sexual arousal, participants with higher sex drive made more errors than participants with lower sex drive. Likewise, hypotheses 5a–c had to be rejected, while Hypothesis 5d was supported: In a state of sexual arousal, participants with a higher propensity to sexually objectify women were more prone to errors indicative of OGC than participants with a lower propensity to do so. See Figs. 4 and 5 for graphs of the moderation effects.

**Table 2:** Moderated hierarchical regressions across mouse-tracking measures

Predictor	∆(X = RT)_T2_				∆(X = MD)_T2_				∆(X = AUC)_T2_				∆(X = ER)_T2_			
	∆*R*^2^	ß	*SE*	*p*	∆*R*^2^	ß	*SE*	*p*	∆*R*^2^	ß	*SE*	*p*	∆*R*^2^	ß	*SE*	*p*
*SDI*																
Model 1	.05			.138	.00			.844	.05			.130	.32			< .001
∆X_T1_		22.65	18.44	.223		0.003	.018	.849		0.053	0.039	.182		0.074	.014	< .001
SDI		23.83	18.44	.200		0.009	.018	.621		0.046	0.039	.250		0.013	.014	.352
Model 2	.00			.600	.01			.865	.03			.104	.18			< .001
∆X_T1_		24.06	18.73	.203		0.004	.018	.824		0.044	0.039	.267		0.054	.013	< .001
SDI		24.32	18.55	.194		0.010	.018	.565		0.053	0.039	.178		0.025	.012	.043
∆X_T1_ * SDI		10.21	19.36	.060		0.012	.019	.531		0.063	0.038	.104		0.061	.012	< .001
*SO*																
Model 1	.10				.00			.935	.05			.125	.32			< .001
∆X_T1_		9.99	19.12	.603		0.004	.018	.227		0.051	0.040	.210		0.071	.015	< .001
SO		45.18	19.12	.021		0.004	.018	.203		0.048	0.040	.237		0.017	.015	.263
Model 2	.00				.02			.583	.01			.527	.21			< .001
∆X_T1_		10.19	19.63	.605		0.007	.018	.686		0.044	0.041	.293		0.035	.014	.013
SO		45.15	19.25	.280		0.002	.018	.922		0.049	0.040	.228		0.012	.012	.340
∆X_T1_ * SO		−0.875	17.24	.960		− 0.028	.021	.180		0.029	0.046	.527		0.055	.010	< .001

*N* = 79. AUC = area under the curve; ER = error rate; MD = maximum deviation; RT = reaction time; SDI = sex drive index; SO = sexual objectification

### Intercorrelations Among Mouse-Tracking Measures and Self-Report Measures

Table 3 shows significant correlations between MT measures at T1 and T2 within the same trial condition (e.g., between AUC_HC_T1_ and AUC_HC_T2_) that demonstrate the reliability (i.e., rank order stability) of the MT paradigm. Furthermore, Table 3 shows the intercorrelations of MT measures at T1 and T2, respectively. In both trial conditions, the high correlations between MD and AUC illustrate the close relationship between these spatial measures. Regardless of the trial condition, RT did not show any significant correlations with other measures at the same measuring time, at least indicating an independent explanatory power of RT.

**Table 3:** Intercorrelations of mouse-tracking measures within the same trial type

	RT_T1_		MD_T1_		AUC_T1_		ER_T1_		RT_T2_		MD_T2_		AUC_T2_	
	HC	LC	HC	LC	HC	LC	HC	LC	HC	LC	HC	LC	HC	LC
MD_T1_	− .13	− .10												
AUC_T1_	− .16	− .13	.94***	.92***										
ER_T1_	.08	− .15	.20	.34**	.35**	.39***								
RT_T2_	.78***	.81***	− .06	− .04	− .12	− .11	.11	− .14						
MD_T2_	− .02	− .11	.75***	.73***	.65***	.68***	.06	.39***	.10	− .03				
AUC_T2_	− .11	− .15	.74***	.68***	.78***	.74***	.35**	.44***	− .02	− .11	.86***	.93***		
ER_T2_	− .08	− .12	.32**	.05	.44***	.11	.74***	.33**	.20	− .12	.22	.27*	.48***	.27*

*N* = 79. AUC = area under the curve; ER = error rate; HC = high conflict condition; LC = low conflict condition; MD = maximum deviation; RT = reaction time. Statistically significant results are in bold

*p < .05; ** *p* < .01; *** *p* < .001

The intercorrelations of the scales (AMMSA, HBI-19, SDI-2, SIS/SES, SOI-R, SOOI, and SUSS) as well as their means, standard deviations, and internal consistencies (αs ≥ .72) can be found in Table 4. As expected, all scales were meaningfully intercorrelated, justifying post hoc the formation of an aggregated sex drive index consisting of SDI-2, SES, and SOI-R to be used as a single moderator. Regarding the two measures of state sexual arousal, descriptively both Arousal_Story_ and Arousal_Now_ correlated positively with all scales (*r*s ≥ .19; see Table 4), indicating a plausible link between dispositional sexual arousability and individual differences in problematic sexuality as measured by the questionnaires. As expected, the two self-report measures of state sexual arousal correlated substantially. Participant age was not related to any self-report measure, nor to the MT difference scores (*p*s ≥ .191). Likewise, participant relationship status was not related to the MT difference scores (*p*s ≥ .284). Descriptively, however, relationship status did exhibit a consistently negative correlation pattern with all assessed self-report measures (*r*s ≤  − .11; see Table 4), echoing prior findings suggesting that men’s current relationship status contributes to predicting their overall perception of women’s sexual exploitability (Lewis et al., 2012).

**Table 4:** Intercorrelations of self-report measures

	*Min*	*Max*	*M*	*SD*	AMMSA	HBI-19	SDI-2	SIS	SES	SOI-R	SOOI	SUSS	SD Index	Arousal_Story_	Arousal_Now_
AMMSA	1.00	4.00	2.03	0.72	(.84)										
HBI-19	1.00	4.25	2.07	0.84	.38***	(.88)									
SDI-2	0.25	6.13	3.92	1.17	.31**	.40***	(.84)								
SIS	1.00	4.25	2.72	0.68	.07	− .12	− .06	(.72)							
SES	1.17	4.67	2.99	0.75	.26*	.40***	.65***	.02	(.78)						
SOI-R	0.44	4.00	2.61	0.80	.37***	.46***	.48***	− .32**	.49***	(.83)					
SOOI	1.44	4.56	2.80	0.81	.52***	.61***	.52***	.03	.50***	.53***	(.83)				
SUSS	0.92	84.15	42.55	21.62	.40***	.41***	.57***	− .02	.49***	.57***	.59***	(.94)			
SD Index	− 2.35	1.36	0.00	0.83	.37***	.51***	.85***	− .14	.86***	.79***	.62***	.65***	(.91)		
Arousal_Story_	2.00	5.00	3.27	1.02	.22	.19	.19	.25*	.40***	.20	.39***	.30**	.32**	( −)	
Arousal_Now_	1.00	4.00	1.96	1.07	.28*	.39***	.36**	.20	.48***	.21	.43***	.46***	.42***	.61***	( −)
RS	0.00	1.00	0.51	0.50	− .16	− .26*	− .11	− .17	− .26*	− .24*	− .21	− .37***	− .24*	− .16	− .20

*N* = 79. AMMSA = Acceptance of Modern Myths about Sexual Aggression Scale; HBI-19 = Hypersexual Behavior Inventory-19; RS = relationship status; SDI-2 = Sexual Desire Inventory-2; SD Index = sex drive index; SIS = Sexual Inhibition Scale; SES = Sexual Excitation Scale; SOI-R = Revised Sociosexual Orientation Inventory; SOOI = Sexual Objectification of Others Inventory; SUSS = Socially Undesirable Sexual Selection Scale. Statistically significant results are in bold. Cronbach’s α in brackets

*p < .05; **p < .01; ***p < .001

### Nomological Network of Overreliance on Global Cues and Problematic Sexuality

In order to test whether there is a nomological network of OGC as assessed by MT measures and common aspects of problematic sexuality as assessed by self-report measures, correlations of these self-report measures with the MT difference scores were examined (Table 5). Since individual differences in problematic sexuality can be considered general correlates of individual differences in appropriate cue interpretation and weighting when assessing another person’s sexual interest, a pattern of positive correlations between MT measures and self-report measures would also indicate convergent validity of the present paradigm.

**Table 5:** Correlations between self-report measures and mouse-tracking difference scores as a function of time

	T1				T2			
	ΔRT	ΔMD	ΔAUC	ΔER	ΔRT	ΔMD	ΔAUC	ΔER
AMMSA	.17	− .03	− .05	.19	− .01	− .18	− .07	.17
HBI-19	.19	.20	.17	.16	.18	.02	.06	.19
SDI-2	.25*	.20	.22	.19	.17	.05	.15	.20
SIS	.17	.01	.01	.07	.11	− .03	.13	.15
SES	.22	.18	.23*	.22*	.19	.05	.11	.18
SOI-R	.03	.15	.18	.26*	.05	.06	.17	.20
SOOI	.34**	.31**	.29**	.43***	.21	.03	.18	.34**
SUSS	.20	.27*	.24*	.23*	.24*	.03	.21	.29**
SD Index	.20	.21	.25*	.27*	.16	.06	.17	.23*
Arousal_Story_	.22	.05	.09	.30**	.26*	.10	.19	.30**
Arousal_Now_	.27*	.24*	.26*	.28*	.41***	.17	.25*	.33**
ReliabilityΔ(α)	.20	.06	.18	.84	.33	.14	.17	.83

*N* = 79. AMMSA = Acceptance of Modern Myths about Sexual Aggression Scale; AUC = area under the curve; ER = error rate; HBI-19 = Hypersexual Behavior Inventory-19; MD = maximum deviation; RT = reaction time; SDI-2 = Sexual Desire Inventory-2; SD Index = sex drive index; SIS = Sexual Inhibition Scale; SES = Sexual Excitation Scale; SOI-R = Revised Sociosexual Orientation Inventory; SOOI = Sexual Objectification of Others Inventory; SUSS = Socially Undesirable Sexual Selection Scale. Δ based on the difference of high conflict trials minus low conflict trials. Statistically significant results are in bold

*p < .05; **p < .01; ***p < .001

Descriptively, all MT difference scores except for ΔMD_T2_ showed theoretically meaningful positive correlations with the self-report measures, with most effect sizes ranging between *r*s of .1 and .3. The most pronounced convergent validity pattern was found for ΔER (both at T1 and T2), whereas the highest correlations were found with the SOOI. Notably, at T1 a much stronger correlational pattern with 41% of the possible correlations being statistically significant was produced (as opposed to only 20% at T2). Mirroring the pattern of convergent validities, the internal consistencies of the MT difference scores (Table 5) were good only for ΔER, whereas the other MT difference scores turned out to be largely unreliable (a common finding for difference scores; Thomas & Zumbo, 2012). Self-reported state sexual arousal at the end of the study (Arousal_Now_) was positively associated with almost every single MT difference score regardless of measuring time.

## Discussion

### Effect of Incongruent Global and Specific Cues

Our first hypothesis stated that there would be differences in RT, MD, AUC, and ER between the two trial conditions HC and LC. In line with their designation, incongruent HC trials were expected to cause some degree of selection difficulty, whereas in congruent LC trials, selection was expected to be rather facilitated. Remarkably, all four MT measures actually differed between the two trial conditions in the predicted manner. Consistent with the results by Smith et al. (2018), a congruency effect was found for both the selection process and the selection outcome, as RT, MD, AUC, and ER were higher in the HC condition, i.e., participants took longer to select an option, mouse trajectories showed more curvature, and more errors were made than in the LC condition. From these findings, it can be tentatively concluded that in situations in which global sexual and specific affective cues co-occur incongruently, men are generally more prone to OGC, even though these two cue types should be regarded as of entirely different informational value for assessing a woman’s current sexual interest in the context of a dyadic social encounter.

It seems plausible that the difficulty in appropriate cue interpretation and weighting in the presence of incongruent global and specific cues represents a ubiquitous effect that occurs in various social settings. This difficulty is also likely to increase further when a global and a specific cue belong to the same category (e.g., affective expression). Saal et al. (1989) demonstrated that in business and academic settings, men tend to overly rely on the global cue of “professional friendliness” when displayed by women, misinterpreting it as (1) being specifically (i.e., selectively) directed at a particular male interaction partner and (2) driven by sexual interest, even if such an interpretation is not supported by context and conversational content (i.e., by specific verbal cues). Given these findings, the general connection between a heightened likelihood of OGC and an elevated risk of sexual harassment becomes notably evident.

### Effect of Sexual Arousal

Our second hypothesis focused on the effect of sexual arousal on OGC. We presumed that in a state of sexual arousal, OGC should be more likely to occur as a result of motivational states affecting decision-making and selection behavior in favor of need-congruent stimuli (e.g., Seibt et al., 2006). As the results show, sexual arousal led to the predicted increase in ER, but not in RT, which instead decreased significantly from T1 to T2 (an unexpected finding reported similarly by Benbouriche et al., 2018). No significant changes were found for MD and AUC. Obviously, mouse movements did not differ spatially between the two measurements, but were executed faster at T2, which at first glance suggests a practice effect and thus a performance improvement. This interpretation, however, is contradicted by the increase in ER, which indicates that the induced state of sexual arousal did, in fact, make it more difficult to choose the normatively correct option, thus also indicating an increase in OGC. For this reason, the decrease in RT might rather be seen as the result of a more disinhibited—and therefore more error-prone—decision behavior in a state of sexual arousal as demonstrated in previous studies (Imhoff & Schmidt, 2014; Wiemer et al., 2023).

### Interaction of Cue Combination and Sexual Arousal

Hypothesis 3 stated an interaction of trial type and time such that the effect of sexual arousal should be more pronounced in HC trials than in LC trials. Analogous to the result pattern of Hypothesis 2, sexual arousal indeed led to a greater increase in ER in the HC condition than in the LC condition, whereas MD and AUC did not differ over time between the two trial types. Contrary to our expectation, RT decreased from T1 to T2 in both trial conditions, with this decrease being even higher in the HC condition. Based on the particularly expressive ER measure, it was thus possible to show that sexual arousal increases the difficulty of making normatively appropriate choices when confronted with incongruent global sexual and specific affective cues in the context of sexual flirting. From this, it can be concluded that apparently for some men, their own visceral/motivational state—and relatedly, their own sexual interest—rather than the information-based assessment of another person’s sexual interest, is the determining factor for sexual selection, which translates into a higher risk for OGC and its detrimental consequences in real social situations.

The significant increase in ER from T1 to T2 observed in the HC condition but not in the LC condition also emphasizes the diagnostic importance of normative errors for the MT paradigm. Contrary to our implicit expectation that differences both between trial types and over time would rather be revealed by the more subtle measures RT, MD, and AUC, the particularly informative ER measure turned out to be the clearest indicator in this respect. This seems encouraging from a methodological point of view, especially as this result suggests that the selection task described above is not susceptible to social desirability bias. While the large relative increase in errors in the sexual arousal condition compared to the baseline condition supports the basic theoretical assumptions of the paradigm and can also be seen as additional evidence for the known disinhibitory effect of sexual arousal (Imhoff & Schmidt, 2014; Wiemer et al., 2023), the low absolute numbers of errors are indicative for the selection task being both comprehensible and well manageable for non-clinical participants. At the same time, the distribution of individual errors in the HC condition at T2 (Fig. 3) points to a subgroup of participants in whom sexual arousal led to a relatively high number of errors (i.e., ≥ 6, indicating that errors occurred in at least 25% of the HC trials at T2). Such a data-driven narrowing of the sample, combined with the pattern of positive correlations between ER and various self-report measures of problematic sexuality, speaks to the forensic-diagnostic potential of the present paradigm.

### Impact of Sex Drive and Sexual Objectification

Hypotheses 4 and 5 stated that both dispositional sex drive and a propensity for sexual objectification would moderate the relationship between sexual arousal and OGC. According to our reasoning, higher sex drive should be associated with higher MT measures, as should a stronger propensity to perceive women primarily in terms of their “sexual usefulness” (Loughnan & Pacilli, 2014). For both potential moderators, although the hypothesized effect could not be confirmed by RT, MD, and AUC, it could be confirmed by ER. Given research findings on the link between men’s sexual objectification of women and an increased likelihood of physical and sexual aggression against women (e.g., Gervais et al., 2014; Vasquez et al., 2017), this result further emphasizes the forensic-diagnostic potential of the MT paradigm. The same applies to the revealed negative impact of high sex drive on appropriate cue differentiation in sexual partner selection, which is consistent with previous findings (Kafka, 2003; Moholy et al., 2015), especially as sex drive is a well-established risk factor for sexual offending (Seto, 2019).

### Association Between Overreliance on Global Cues and Problematic Sexuality

In addition to our hypotheses, we explored our conjecture of a nomological network of OGC and various aspects of problematic sexuality. To this end, Pearson correlations of the MT difference scores with self-report measures assessing problematic sexual attitudes, behaviors, experiences, and motivations were conducted, the latter of which can be considered general correlates of difficulties in appropriate cue interpretation and weighting when assessing another person’s sexual interest. On a descriptive level, all MT difference scores except for ΔMD_T2_ showed theoretically meaningful positive correlations with most of the self-report measures applied in this study (i.e., AMMSA, HBI-19, SDI-2, SIS/SES, SOI-R, SOOI, and SUSS), thereby indicating convergent validity of the present paradigm. The strongest convergent validity pattern at both measuring times was found for ΔER, which included the two measures of state sexual arousal (Arousal_Story_, Arousal_Now_), while also showing good internal consistencies. Descriptively, both measures of state sexual arousal correlated positively with all self-report measures and all MT measures, thus pointing to the importance of dispositional sexual arousability in the context of both problematic sexuality and OGC. Taken together, these correlational patterns confirm a nomological network of various aspects of problematic sexuality, higher sexual arousability, and OGC. From a methodological perspective, these distinct patterns can be considered a strong safeguard against artificial or coincidental single correlations driven by outliers or spurious effects. Surprisingly, all meaningful correlations between MT difference scores and self-report measures could already be found at T1, suggesting that a single measurement might be sufficient from a diagnostic point of view. Apart from that, this finding highlights the usefulness of difference scores as indicators of OGC as conceptualized in the current study.

### Limitations

The findings presented in this study are inevitably subject to some limitations. First, the comparatively small sample size limits the interpretability of the results. At the same time, the within subjects-design used is superior to a between-subjects design in terms of power under the assumption of normally distributed data (Maxwell & Delaney, 2004), so preliminary conclusions can certainly be drawn from the results that warrant further research. Nevertheless, a future increase in sample size would be necessary to potentially support the findings of the present study with higher statistical power, which is especially true for the moderating effects.

Another potential drawback of our study could be seen in the lack of a (non-aroused) control group at T2, which would have served to control for possible learning effects or test motivation effects. However, we minimized the risk of overlooking such effects by the 2 × 2 design of our paradigm: While a learning effect is ruled out by the performance decline observed in the HC trials between T1 and T2 (as evidenced by the increase in ER), a fatigue or test motivation effect is contradicted by the fact that the same number of errors were made in the LC trials at T1 and T2, respectively, strongly indicating that there was no general response bias on the part of the participants. Thus, the design of our experiment rendered a control group largely unnecessary.

We used a convenience sample according to the WEIRD criteria established by Henrich et al. (2010), i.e., participants came from a population that can be characterized as western, educated, industrialized, rich, and democratic, with nearly 70% being university students. While such sample composition usually implies a lack of generalizability of results (Hanel & Vione, 2016), it can be considered methodologically promising in the case of the present study. Despite the non-representative composition of the sample—especially with regard to criterion-related attitudes toward sexuality, sexual consent, and women in general, as well as executive functioning, educational level, personality traits, and socioeconomic status—the collected data already showed enough systematic variance to draw conclusions about the relationship between our experimentally manipulated factors and individual differences in both propensity for OGC and self-reports of problematic sexuality. It is thus to be expected that the present effects would be all the more evident in a larger and more mixed sample of men with more diverse attitudes, social backgrounds, and sexual behaviors and motivations, especially with a future focus on populations with increased risk for sexual (re)offending.

Trials in the HC condition consisted of two images of the same woman, each featuring a combination of incongruent cues (i.e., Casual × Flirting and Sexy × Rejecting). Following ratings for perceived sexual attractiveness per image, only those image pairs were used in the study for which the difference between the two attractiveness rating scores was close to zero in order to ensure that both images conveyed a similar level of sexual attraction (see ES1 for details). Instead, however, it would have been perfectly reasonable (and probably closer to reality) to maximize selection conflict by using those image pairs for the HC condition in which the sexual attraction of the Sexy × Rejecting image clearly exceeded that of the Casual × Flirting image, thereby potentially increasing baseline occurrence of OGC. Therefore, the more conservative stimulus selection performed for the current study might have led to smaller effect sizes for all MT measures. Especially concerning ER, a floor effect was observed, as most participants made few or no errors not only at T1, when it was expected, but also at T2. Since ER proved to be a particularly informative indicator of OGC in the present paradigm, it would be methodologically desirable to avoid or mitigate such a floor effect in the future. Another advantage of using image pairs with more varying attractiveness-related difference scores would be the opportunity to meaningfully correlate these differences scores with MT measures, further validating the conflict-inducing effect of the HC condition (in addition to the *t*-tests conducted for this purpose; see Stimuli and Trial Types section) at the level of single trials.

### Strengths

Despite the above limitations, the present study is characterized by several strengths of design and data collection. First, the pictorial stimuli used were created specifically to meet predefined conceptual requirements. These requirements allowed for the two trial conditions HC and LC, thus ensuring the functionality of the selection task. Moreover, the stimuli can be expected to increase the ecological validity of the paradigm, as we afforded our models as much individual freedom as possible in interpreting—at the same time well-defined—categories of (1) clothing and (2) facial expression. Effect-related properties of the stimuli were validated prior to the study in a multi-step procedure (see ES1 for details). This extensive preliminary work is a clear advantage over comparable studies that rely on less specific/less standardized stimuli to answer their questions, which also means that confounding variables (e.g., body language, differences in attractiveness on multiple levels) cannot be controlled for to the same extent.

Smith et al. (2018) examined the relationship between men’s subjective ratings of women’s sexual interest and their propensity to engage in sexually aggressive behavior. While for this purpose Smith et al. (2018) presented one stimulus per trial, which participants were asked to assign to one of two categories (“sexually interested” or “rejecting”), in the current study the choice was not between two semantic options, but between two simultaneously presented images. We see the advantage of such a two-image design (1) in that there are no detours due to image-to-text-abstraction, as participants’ selection conflict arises directly from two competing visual stimuli depicting the same woman. Moreover, as a direct result of the two-image design, (2) the choice to be made is not based on absolute, i.e., simplified categories (flirting or rejecting), but on a relative instruction (“Click on the woman who is more likely to flirt with you right now”) by which, in addition, (3) the concept of rejection is not explicitly introduced to the participants, allowing for more authentic decisions.

The selection task takes the form of a flirting scenario and is thus embedded in a social context characterized by its everyday relevance and comprehensibility. The task does not require any particular abstraction from the participants, and since the instructions used are brief and simple, they should prove easy to understand even in target populations with lower levels of education (e.g., forensic samples). Induction of sexual arousal was achieved noninvasively by means of an erotic audio story, i.e., without recourse to explicit visual material. Spontaneous positive feedback suggests that the study did not elicit substantial reactance among participants. The time required for both parts of the selection task and listening to the audio story in between is about 12 min, which is within reason for a diagnostic task. Finally, the in-person survey, as opposed to a more easily conducted online survey, ensured that the participants in this pilot study all used the same equipment, complied with the general instructions, and, in particular, actually listened to the erotic audio story, which was crucial for the induction of sexual arousal between the two measurements.

## Outlook

### Research Desiderata

From the above, suggestions for improving the present paradigm can be derived for future research. Since one focus of this study was to maximize ecological validity, effect-related properties of the stimuli were kept within a range that corresponds to real social settings (e.g., authentic clothing). This closeness to everyday life is at the expense of effect maximization, which could be achieved by means of several—also combinable—approaches. The main consequence of such effect optimization should be an increase in selection conflict experienced by future participants, which in turn should lead to greater variability in MT measures. This could be done, for example, by enhancing clothing contrasts (e.g., by increasing skin display and clothing tightness in the Sexy clothing condition) or by adding information in the form of iconic or semantic cues (as successfully done by Lim et al., 2018) that might alter the perceived sexual attractiveness of the depicted women (e.g., cues with regard to purported [non]promiscuous behavior; see Stewart-Williams et al., 2016).

As a prerequisite for measuring propensity for OGC using the present paradigm, participants must be sexually aroused at T2. For this purpose, an erotic audio story was used in the current study. The manipulation check question about its effect showed that about 8% of the participants claimed to have found the story not to be sexually stimulating at all. Although this is already a fairly small proportion (which may even have been increased by social desirability bias), a more reliable method of inducing sexual arousal in male participants, e.g., by using audiovisual erotic stimuli, would certainly be beneficial. This is all the more true since participants were found to be less aroused in response to erotic audio-only stimuli than audiovisual stimuli once they became familiar with the procedure (Golde et al., 2000). However, to avoid compromising the applicability of the MT paradigm in practical contexts, a trade-off between reliability and invasiveness of the method used for inducing sexual arousal should be considered.

In a broader context, it is also conceivable to measure additional or alternative independent variables (i.e., aside from sexual arousal) using the MT paradigm, such as alcohol or heat. As previous research has shown, alcohol consumption in men leads to misjudgments regarding women’s sexual interest (Benbouriche et al., 2018; Farris et al., 2010) and adds to the risk of sexual violence perpetration among men already predisposed to sexual aggression (Abbey, 2011). Likewise, it has been demonstrated that higher temperatures are associated with an increased number of sexual assaults (Anderson, 1989; Field, 1992). These effects might be detectable via the present paradigm by manipulating participants’ alcohol consumption and/or the ambient temperature, with OGC as assessed by MT measures remaining the central outcome variable. The paradigm’s within-subjects design would prove particularly advantageous here, as a comparison between T1 and T2 would allow for the capture of intraindividual changes occurring after exposure to the factor(s) of interest and, thus, to focus on *when* some men exhibit sexually problematic behavior, rather than on *who* displays such behavior (see Abbey, 2011).

### Theoretical Implications: Different Causes for Overreliance on Global Cues

Male sexual misperception of women’s sexual interest has been thoroughly studied under the premise that for consensual sexual acts to occur, another person’s current (lack of) sexual interest must be correctly perceived (e.g., Perilloux et al., 2012; Treat et al., 2016, 2017). According to our view, OGC in the context of sexual misperception can be explained at least partially by a failure to recognize the different informational value of global sexual and specific affective cues. But even if this different informational value in assessing another person’s (non)sexual intentions is correctly discerned, OGC may still occur as a result of insufficient sexual self-control. Exerting self-control proves necessary when a subjectively experienced conflict arises in choosing between mutually exclusive options, where one option corresponds to a spontaneous (often pleasure-oriented, yet in some respect detrimental) preference, while the other option is consistent with a more long-term (often normative) objective that is rationally prioritized (e.g., Gillebaart, 2018). These mutually exclusive options, however, may well have been adequately assessed in terms of their respective meanings and possible consequences. Thus, OGC in the context of sexual self-control occurs not because of an inadequate interpretation of global and specific cues, but because the attraction of the global sexual cue—which may be further enhanced by a state of sexual arousal—cannot be resisted despite an “honest attempt” to adhere to a social or personal norm or an instruction demanding the opposite. The absence of such an “honest attempt” constitutes the third theoretical reason for OGC, which we see in egocentric hedonism (i.e., a strong emphasis on one’s own [sexual] satisfaction) on the part of the selecting male. In this case, it does not matter whether the woman’s rejecting or consenting attitude is correctly perceived, since this aspect has no relevance for the man’s selection, which is instead based mainly or exclusively on a stable hedonic preference for sexual cues that goes subjectively unchallenged by opposing interests and social norms. In the perceived absence of a competing (i.e., normative) option, no conflict occurs, so self-control is not a factor in this scenario. Obviously, such a sexual selection strategy greatly increases the likelihood of detrimental consequences of OGC, since no subsequent behavioral adjustment can be expected, neither from a corrected perception nor from a renewed effort of will.

On the surface, it may seem that a mere distinction into sexual selection based on either global or specific cues should prove sufficient in terms of experimental measurability, especially for eventual application in the forensic field, where (predicted) misconduct is judged in absolute terms, i.e., based on its adverse effect on others. In fact, however, it would undoubtedly be beneficial for both forensic and diagnostic/therapeutic purposes to distinguish between different mechanisms that underlie OGC as assessed by sexual selection tasks. While we assume that in our experiment participants’ errors at T2 were likely not due to sexual misperception—as recognition and normatively correct interpretation of global and specific cues at T1 has been almost entirely successful—it remains unclear whether errors at T2 were rather due to a failure of sexual self-control or whether breakthroughs of egocentric hedonism also occurred at this point. One way to distinguish between these latter two possible causes of OGC might be to analyze the mouse trajectories of error trials. We hypothesize that errors resulting from a lack of sexual self-control would be associated with rather curved trajectories (since there has usually been an unsuccessful attempt to resist the attraction of the sexual cue and to target the normatively correct option), whereas errors stemming from egocentric hedonism would be found at the end of rather straight trajectories (since a non-conflicting decision has been made based solely on personal preference for the sexual cue). A prerequisite for such an analysis would be a sufficiently high error baseline at T2, which could be achieved, among other things, by (1) enhancing the attraction of the sexual cue, (2) increasing participants’ level of sexual arousal, and/or (3) reducing the response window for the forced selection.

## Conclusion

The present study was able to show that an MT selection task is a promising approach for the (indirect) behavioral measurement of male OGC. Both manipulated factors—i.e., trial type and measuring time—proved to be of additional methodological value. In particular, the induction of sexual arousal prior to the second measuring time (1) highlighted the importance of ER as an indicator of OGC in the present paradigm and (2) allowed to demonstrate the moderating function of two constructs associated with socially problematic sexual behavior, namely dispositional sex drive and sexual objectification.

The distinct advantage of MT is seen in that recording the movement trajectories of a computer mouse makes it possible to trace the progression of selection processes up to their respective outcomes in real time (Freeman & Ambady, 2010a). MD and AUC, as numerical indicators of the spatial course of a trajectory, represent the procedure’s genuine measures. While some researchers highlight the novel utility of MT and emphasize its potential (e.g., Kieslich & Henninger, 2017; Wulff et al., 2019), other voices are critical and express concerns about a possible redundancy of MT’s genuine measures compared to more conventional ones, such as RT and ER (e.g., Bartels et al., 2019).

As for the results of the current study, MD and AUC were indeed found to be considerably less conclusive than RT and ER. However, not least due to the limited sample size, it currently remains unclear whether RT and ER really provide more reliable estimates or whether MD and AUC are mere epiphenomena of the measurement (Bartels et al., 2019), so that one could possibly forego the use of a computer mouse (as well as a comparatively intricate data analysis) in favor of a keyboard-based approach. Also, both spatial measures may still prove crucial for differentiating causal mechanisms underlying OGC and thus provide important diagnostic information, e.g., in the context of therapeutic interventions and risk assessments. Finally, it cannot be ruled out that a keyboard-based approach would not yield results of the same quality even if MD and AUC should indeed turn out to be informatively redundant, as the moving of a computer mouse as such (due to the thereby given possibility to change one’s mind, and even multiple times) might be a necessary precondition for producing errors of diagnostic and forensic significance.

### Supplementary Information

Below is the link to the electronic supplementary material.

Supplementary file1 (DOCX 41 kb)

## Data Availability

Upon reasonable request, the data and material can be obtained from the corresponding author.

## Footnotes

1 While there are flirting motivations other than initiating sexual contact, men tend to associate flirting cues with sexual intentions (Henningsen, 2004). Since the present study specifically aimed to measure heterosexual men’s responses to female flirting cues in a state of sexual arousal, it focused on men’s dominant view of flirting cues as signs of sexual interest. Moreover, for our flirting condition (see Stimuli and Trial Types section), female models were explicitly instructed to flirt sexually, i.e., to flirt with someone they found sexually attractive.

2 A nomological network is a theoretical framework representing the systematic relationships between a construct, its observable indicators, and other constructs of a specific domain (Cronbach & Meehl, 1955).

3 One further participant had to be removed due to clearly outlying MT results (see Statistical Analyses section).

4 One item from the original scale was excluded due to a lack of content fit (i.e., reference to prostitution).

## Code Availability

Upon reasonable request, the code can be obtained from the corresponding author.
