# Supplementary material for: A Dress Is Not a Yes: Towards an Indirect Mouse-Tracking Measure of Men’s Overreliance on Global Cues in the Context of Sexual Flirting
Source: Arch Sex Behav. 2024 Feb 7;53(6):2063–82. doi: 10.1007/s10508-023-02798-x (PMC11176100; doi:10.1007/s10508-023-02798-x)
Supplement: Supplementary file 1 — Supplementary file1 (DOCX 41 kb) [file 10508_2023_2798_MOESM1_ESM.docx]

**Electronic Supplement**

**ES1: Conception, Creation, and Validation of the Stimuli**

**Models**

Models were 64 females whose ages ranged from 18 to 29 years (*M* = 22.33, *SD* = 2.42, *Mo.* = 23) and whose heights ranged from 1.58 m to 1.81 m (*M* = 1.69, *SD* = 0.05, *Mo.* = 1.68). While hairstyles (e.g., dreadlocks, particularly long or short hair), inconspicuous jewelry, and tattoos were not controlled for, glasses were excluded (1) to prevent light reflections in the photographs, (2) because they would be too easy a discriminatory cue if models were to switch between glasses and contact lenses for different outfits (see below), and (3) to avoid confounding effects on participants’ perceptions of attractiveness (Leder et al., 2011; Terry & Hall, 1989).

**Photo Shoots**

Each model was to bring two outfits of their own choice, which despite their desired individuality had to be clearly assignable to two distinct categories we had defined in advance (i.e., Casual and Sexy).^[[1]](#footnote-1)^ The Casual outfit had to be everyday clothes that were neither tight nor revealing—clothing in which one would, for example, “visit a good friend to hang out together” or “spontaneously run an errand in town.” A line was drawn at a type of clothing that might be perceived as too careless and unkempt, i.e., clothing that one would not wear in public. Makeup was optional in the Casual condition but had to be natural. In stark contrast to the Casual outfit, clothing style and accessories of the Sexy outfit were supposed to match “having a night out with the general intention to flirt.” For this purpose, tight-fitting and revealing clothing was recommended (but not strictly required, since authenticity was the key criterion for both clothing conditions to maximize ecological validity), as well as conspicuous makeup (e.g., lipstick, rouge, mascara).

After collecting basic demographic data, photographs were taken of the models showing two facial expressions (i.e., Flirting and Rejecting) in each of the two outfits. To evoke facial responses as authentic as possible, models were instructed to imagine two social situations. For the Flirting expression, they were asked to imagine a situation in which they would like to persuade a person they are sexually interested in to approach them by using a flirtatious facial expression. In contrast, for the Rejecting expression, they should imagine a situation in which they want to discourage an obnoxious person from approaching them any further by using a rejecting facial expression. Following Ebner et al. (2010), both facial expressions should be shown intensely but as naturally as possible.

Since only the outfit (i.e., clothing, accessories, hairstyle, makeup) and facial expression cues were to be varied for the experiment, models were instructed not to change their posture and head position between shots and to pose upright with legs neither bent nor too far apart, arms comfortably at their sides, and face forward. This instruction was based on findings that body language influences the perception of emotional facial expressions to a large extent (Van den Stock et al., 2007). Also, since recognition of the model’s facial expressions should be possible even when stimuli were to be presented briefly or reduced in size, the shooting angle was deliberately chosen to create a slight distortion of proportions in favor of the upper half of the body, especially the face.

**Initial Image Selection and Image Editing**

In the course of the 64 photo shoots, more than 3,500 photographs^[[2]](#footnote-2)^ were taken using a digital camera. After reviewing this material several times, the authors selected four images per model containing each of the outfit × facial expression combinations (i.e., Casual × Flirting, Casual × Rejecting, Sexy × Flirting, Sexy × Rejecting), resulting in a set of 256 images. It was considered essential for selection that an image simultaneously met the general criteria (i.e., posture, limb position, gaze direction), the cue-specific criteria (i.e., prima facie achieving the desired impression of outfit and facial expression), and the technical requirements (i.e., sharpness, color effect, lighting).

As background for the photo shoots, a green screen was used to facilitate subsequent cropping of the images using standard photo editing software. First, the green background was removed and replaced with a uniform white background. Then the images were cropped and the respective distances between the top, bottom, and side edges of the depicted bodies and the margins of the images were standardized.

**Sexual Attractiveness Rating**

In order to obtain the two parallel sets required for the intended experiment—which should be as similar as possible in terms of both mean and distributed sexual attractiveness values of the included images—all images were rated for perceived sexual attractiveness on a five-point scale by 12 naive, heterosexual male raters between 23 and 56 years (*M* = 30.58, *SD* = 8.77). The written instruction read:

You will now be shown images of different women. Please spontaneously rate how sexually attractive you find each woman. Please rate each image independently of the other images. You can give your rating from 1 for *not sexually attractive at all* to 5 for *very sexually attractive*.

The mean sexual attractiveness of the whole stimulus set was *M* = 2.61 (*SD* = 0.27). With regard to the 256 individual images, mean sexual attractiveness values ranged from *M* = 1.17 to *M* = 4.17.

**High vs. Low Conflict Condition**

In each trial of the experiment, two images of the same woman were to be shown simultaneously, thus avoiding a confounding effect of differences in perceived sexual attractiveness that would most likely result from a presentation of two different women. The chosen design was predicated on the assumption that heterosexual men perceive both a sexually suggestive outfit and a flirtatious facial expression on the part of women as (sexually) appealing (e.g., Abbey et al., 1987; Lau, 1982). At the same time, it was reasonable to assume that both inconspicuous clothing and a rejecting facial expression would not be perceived as equally appealing. Instead, these cues could be expected to be perceived as rather neutral (in the case of the casual outfit) or even as negative (in the case of the rejecting facial expression; e.g., Grammer et al., 2004; Moore, 1998).

Our basic premise for evoking conflicts was that the respective two outfit and facial expression cues would be perceived as sexually appealing to different degrees (i.e., Casual < Sexy, Rejecting < Flirting), so that their (in)congruent combinations would lead to the effects of individual cues per image either adding up (Sexy & Flirting; Casual & Rejecting) or somewhat counteracting each other (Sexy vs. Rejecting; Casual vs. Flirting). This assumption was confirmed by *t*-tests. Consistent with our predictions, images of the Flirting condition were rated as more sexually attractive than images of the Rejecting condition with *t*(63) = 10.59, *p* < .001, *d*_z_ = 1.32. Likewise, images of the Sexy condition were rated as more sexually attractive than images of the Casual condition with *t*(63) = 7.49, *p* < .001, *d*_z_ = 0.94.

Crucially, we also found that comparing the two mixed cue conditions Sexy × Flirting (SeFl) and Casual × Rejecting (CaRe), the mean difference in perceived sexual attractiveness was particularly clear with *t*(63) = 12.32, *p* < .001, *d*_z_ = 1.54. This did not come as a surprise, since the depicted woman displayed congruent cues in both images: both positive cues in the SeFl image and both non-positive cues in the CaRe image. Hence, we expected future participants to show a clear preference for the SeFl image in these pairs. Due to this expected clear preference, we designated the CaRe × SeFl image combination as the low conflict (LC) trial condition of our experiment. In contrast, the high conflict (HC) trial condition was based on incongruent cue combinations. In both images of this trial condition, the depicted woman displayed one positive and one non-positive cue, resulting in the two mixed cue conditions Sexy × Rejecting (SeFr) and Casual × Flirting (CaFl). As indicated by a *t*-test with *t*(63) = 0.91, *p* = .184, *d*_z_ = 0.11, these cue conditions were not perceived differently in terms of their overall sexual attractiveness. Consequently, their juxtaposition, i.e., the SeFr × CaFl image combination, could be expected to cause a selection conflict of some degree on the part of future participants.

**Interrater Agreement on Outfits and Facial Expressions**

Although the general effectiveness of the stimuli had already been confirmed by the *t*-tests described above, it was necessary to verify for each individual image whether it would have the intended effect on an untrained observer. For this purpose, all 256 images were rated on two aspects by six further naive, male, heterosexual raters between 23 and 34 years of age (*M* = 27.67, *SD* = 3.45). Since two images were to be shown simultaneously in each trial of the experiment, having relative rather than absolute ratings of both the outfits and the facial expressions was deemed sufficient. To this end, two images of the same model were compared in each rating trial, with 64 trials showing image pairs in the HC condition and another 64 trials showing image pairs in the LC condition. Both the order of the two conditions and the position (left/right on the screen) of each image per trial were randomized. First, the outfits were rated. The question to be answered was, “Which outfit do you find more sexy?” Then, the facial expressions were rated. The question now read, “Which facial expression seems more flirtatious to you?”

Interrater reliability was determined using Krippendorff’s α for nominal data (Hayes & Krippendorff, 2007). Results indicated high to very high levels of agreement for both the outfits (*r*_α_ = .82) and facial expressions (*r*_α_ = .93). In all cases of full rater agreement, the rating matched with the intended impression of either clothing style or facial expression, i.e., the Sexy outfit was indeed perceived as more sexy than the Casual outfit and the Flirting expression perceived as more flirtatious than the Rejecting expression. There was no complete agreement among raters in only one of the 128 comparisons of facial expressions (0.8%) and in five of the 128 comparisons of outfits (3.9%). Since in these cases the majority of ratings were still consistent with the intended impression, no image pairs were excluded from the stimulus set at this point.

**Final Image Selection and Generation of Parallel Sets**

In order to maximize the conflict participants should face when choosing between the two images of an HC trial, the images of such a pair had to be similar in terms of sexual attraction. If the difference between the two mean attractiveness values were too large, this would result in an unwanted bias in favor of one of the two images, and the decision to be made would then not be based on the intended criterion, namely individual preference for either global sexual or specific affective cues. Such similarity was tested for by calculating difference scores for each image pair (∆ *M*_SeRe_, *M*_CaFl_). If the difference of the two mean attractiveness values of an HC image pair resulted in a score that was not sufficiently close to zero—the tolerance limit was conservatively set to |0.5|—the image pair in question was removed from the set. This was the case for 16 pairs, so that 48 pairs of the HC condition remained in the set, whose pairwise difference scores ranged from −0.42 to 0.42 (*M* = 0.24, *SD* = −0.03). In a next step, the mean attractiveness value was calculated for each of the remaining image pairs. In the HC condition, these pairwise mean attractiveness values ranged from 1.29 to 3.75 (*M* = 2.50, *SD* = 0.65). As expected, a *t*-test conducted on the reduced HC set (CaFl vs. SeRe) yielded a clearer result than before with *t*(47) = 0.22, *p* = .414, *d*_z_ = 0.04, further indicating the absence of a group difference between these two incongruent cue combinations.

As with the HC condition, a difference score was calculated for each image pair in the LC condition (∆ *M*_SeFl_, *M*_CaRe_). This time, positive difference scores were expected since images with SeFl combination should have higher mean sexual attractiveness values than images with CaRe combination. To equalize the number of trials from both conditions, 16 image pairs had to be excluded again, namely the pairs whose difference scores were closest to zero, i.e., the least non-conflicting pairs. The pairwise difference scores of the remaining LC image pairs ranged from 0.25 to 1.42 (*M* = 0.66, *SD* = 0.32), while their pairwise mean attractiveness values ranged from 1.63 to 3.92 (*M* = 2.69, *SD* = 0.61). As anticipated, a *t*-test conducted on the reduced LC set (CaRe vs. SeFl) yielded a more pronounced result than before with *t*(47) = 14.15, *p* < .001, *d*_z_ = 2.04, further emphasizing the group difference between these two congruent cue combinations.

Three of the 16 image pairs excluded from the LC set featured a model whose images had already been excluded from the HC set; each model of the other 13 image pairs excluded per condition appeared on images of the opposite condition. The fact that the majority of women were to be shown in both parallel sets was quite desirable, as it would allow for even better masking of the experimentally crucial HC condition by visually similar trials of the LC condition. Each image would be presented only once during the entire experiment.

In order to form two parallel sets using the 2 × 48 selected image pairs, the difference scores explained above were first put into two ranking orders—separate for the HC and LC condition—and divided according to an alternating pattern (i.e., ABBAAB). In this way, two matching subsets A and B with 24 image pairs each were formed for each trial condition which were already very similar with respect to both their mean difference score and the ranges of pairwise difference scores (see Tables ES1 and ES2). In a next step, several image pairs were swapped between the subsets of the same condition to provide additional parallelization based on the mean attractiveness value per subset and also to approximate the ranges of pairwise mean attractiveness values in the subsets (see Tables ES1 and ES2). Finally, parallelization was increased by comparable frequency distributions of—respectively categorized—pairwise difference scores and pairwise mean attractiveness values, with additional attention paid here to sufficiently similar category means (see Tables ES3 and ES4). This multi-stage approach resulted in remarkably high similarity between the two parallel sets in terms of effect-related properties. The first parallel set is composed of the A subsets of the two conditions; analogously, the second parallel set is composed of the two B subsets.

**References**

Abbey, A., Cozzarelli, C., McLaughlin, K., & Harnish, R. J. (1987). The effects of clothing and dyad sex composition on perceptions of sexual intent: Do women and men evaluate these cues differently? *Journal of Applied Social Psychology*, *17*(2), 108–126. https://doi.org/10.1111/j.1559-1816.1987.tb00304.x

Ebner, N. C., Riediger, M., & Lindenberger, U. (2010). FACES: A database of facial expressions in young, middle-aged, and older women and men: Development and validation. *Behavior Research Methods*, *42*(1), 351–362. https://doi.org/10.3758/brm.42.1.351

Grammer, K., Renninger, L. A., & Fischer, B. (2004). Disco clothing, female sexual motivation, and relationship status: Is she dressed to impress? *Journal of Sex Research*, *41*(1), 66–74. https://doi.org/10.1080/00224490409552214

Hayes, A. F., & Krippendorff, K. (2007). Answering the call for a standard reliability measure for coding data. *Communication Methods and Measures*, *1*(1), 77–89. https://doi.org/10.1080/19312450709336664

Landwehr, I., & Mundloch, K. (2023). *PICSA: A pictorial stimulus set of (in)congruent sexual and affective cues in women* [Manuscript in preparation]. Department of Psychology, Johannes Gutenberg University Mainz.

Lau, S. (1982). The effect of smiling on person perception. *The Journal of Social Psychology*, *117*(1), 63–67. https://doi.org/10.1080/00224545.1982.9713408

Leder, H., Forster, M., & Gerger, G. (2011). The glasses stereotype revisited. *Swiss Journal of Psychology*, *70*(4), 211–222. https://doi.org/10.1024/1421-0185/a000059

Moore, M. M. (1998). Nonverbal courtship patterns in women: Rejection signaling: An empirical investigation. *Semiotica*, *118*(3-4). https://doi.org/10.1515/semi.1998.118.3-4.201

Terry, R. L., & Hall, C. A. (1989). Affective responses to eyeglasses: Evidence of a sex difference. *Journal of the American Optometric Association*, *60*(8), 609–611.

Van den Stock, J., Righart, R., & de Gelder, B. (2007). Body expressions influence recognition of emotions in the face and voice. *Emotion*, *7*(3), 487–494. https://doi.org/10.1037/1528-3542.7.3.487

**Table ES1**

*Mean Difference Scores and Mean Sexual Attractiveness Values of the Subsets*

|  | High Conflict | |  | Low Conflict | |
| --- | --- | --- | --- | --- | --- |
|  | Difference | Attractiveness |  | Difference | Attractiveness |
| Subset A | −0.04 (0.23) | 2.50 (0.67) |  | 0.65 (0.31) | 2.69 (0.64) |
| Subset B | −0.02 (0.24) | 2.50 (0.63) |  | 0.66 (0.32) | 2.70 (0.59) |

*Note*. Standard deviations in brackets.

**Table ES2**

*Ranges of Pairwise Differences Scores and Pairwise Mean Sexual Attractiveness Values in the Subsets*

|  | High Conflict | |  | Low Conflict | |
| --- | --- | --- | --- | --- | --- |
|  | Differences | Attractiveness |  | Differences | Attractiveness |
| Subset A | [−0.42; 0.42] | [1.29; 3.67] |  | [0.33; 1.42] | [1.63; 3.92] |
| Subset B | [−0.42; 0.42] | [1.46; 3.75] |  | [0.25; 1.33] | [1.83; 3.88] |

**Table ES3**

*Frequency Distribution of Pairwise Differences Scores in the Subsets*

| High Conflict | | | | |  | Low Conflict | | | | |
| --- | --- | --- | --- | --- | --- | --- | --- | --- | --- | --- |
| LL; UL | *f*(x)_A_ | *M*_A_ | *f*(x)_B_ | *M*_B_ |  | LL; UL | *f*(x)_A_ | *M*_A_ | *f*(x)_B_ | *M*_B_ |
| [−0.50; −0.26] | 4 | −0.38 | 4 | −0.38 |  | [0.25; 0.49] | 9 | 0.38 | 9 | 0.37 |
| [−0.25; −0.11] | 5 | −0.18 | 5 | −0.20 |  | [0.50; 0.74] | 7 | 0.55 | 7 | 0.56 |
| [−0.10; 0.10] | 9 | −0.03 | 8 | 0.02 |  | [0.75; 0.99] | 3 | 0.83 | 2 | 0.88 |
| [0.11; 0.25] | 4 | 0.21 | 4 | 0.19 |  | [1.00; 1.24] | 4 | 1.10 | 4 | 1.08 |
| [0.26; 0.50] | 2 | 0.42 | 3 | 0.39 |  | [1.25; 1.50] | 1 | 1.42 | 2 | 1.29 |

*Notes*. A = subset A; B = subset B; LL = lower limit; UL = upper limit; x = pairwise difference scores within the interval specified for each condition in the respective first column.

**Table ES4**

*Frequency Distribution of Pairwise Mean Sexual Attractiveness Values in the Subsets*

| High Conflict | | | | |  | Low Conflict | | | | |
| --- | --- | --- | --- | --- | --- | --- | --- | --- | --- | --- |
| LL; UL | *f*(x)_A_ | *M*_A_ | *f*(x)_B_ | *M*_B_ |  | LL; UL | *f*(x)_A_ | *M*_A_ | *f*(x)_B_ | *M*_B_ |
| [1.00; 2.00] | 7 | 1.70 | 7 | 1.71 |  | [1.00; 2.00] | 4 | 1.72 | 3 | 1.85 |
| [2.01; 2.50] | 6 | 2.31 | 6 | 2.38 |  | [2.01; 2.50] | 6 | 2.25 | 7 | 2.23 |
| [2.51; 3.00] | 5 | 2.78 | 6 | 2.87 |  | [2.51; 3.00] | 5 | 2.79 | 5 | 2.70 |
| [3.01; 3.50] | 4 | 3.28 | 4 | 3.22 |  | [3.01; 3.50] | 7 | 3.22 | 7 | 3.20 |
| [3.51; 4.00] | 2 | 3.63 | 1 | 3.75 |  | [3.51; 4.00] | 2 | 3.79 | 2 | 3.81 |

*Notes*. A = subset A; B = subset B; LL = lower limit; UL = upper limit; x = pairwise mean attractiveness values within the interval specified for each condition in the respective first column.

**ES2: Preliminary Validation of the Socially Undesirable Sexual Selection Scale (SUSS)**

Based on a convenience sample of *N* = 266 community participants (74% women), the 13-item Socially Undesirable Sexual Selection Scale (SUSS; Schmidt, 2023) yielded an internal consistency of α = .89. In terms of scale discriminant validity the SUSS was not associated with participant sex, age, extraversion (Rammstedt & John, 2005), and relationship status (│*r*s│ ≤ .09, *p*s > .05), whereas sex drive-related measures such as sexual preoccupation (Wiederman & Allgeier, 1993), solitary and dyadic sex drive (SDI-2; Spector et al., 1996), all sociosexual orientation (SOI-R; Penke & Asendorpf, 2008) subscales, and total sexual outlets revealed positive correlations ranging from *r* = .17 to *r* = .37 (*p*s < .01) and a measure of general (not sexuality-related) self-control (SRSQ; Imhoff et al., 2014) showed a small negative correlation of *r* = −.16, *p* < .01, corroborating the scale’s convergent validity.

**References**

Imhoff, R., Schmidt, A. F., & Gerstenberg, F. (2014). Exploring the interplay of trait self-control and ego depletion: Empirical evidence for ironic effects. *European Journal of Personality*, *28*(5), 413–424. https://doi.org/10.1002/per.1899

Penke, L., & Asendorpf, J. B. (2008). Beyond global sociosexual orientations: A more differentiated look at sociosexuality and its effects on courtship and romantic relationships. *Journal of Personality and Social Psychology*, *95*(5), 1113–1135. https://doi.org/10.1037/0022-3514.95.5.1113

Rammstedt, B., & John, O. P. (2005). Kurzversion des Big Five Inventory (BFI-K): Entwicklung und Validierung eines ökonomischen Inventars zur Erfassung der fünf Faktoren der Persönlichkeit [Short version of the Big Five Inventory (BFI-K): Development and validation of an economic inventory for assessment of the five factors of personality]. Diagnostica, 51(4), 195–206. https://doi.org/10.1026/0012-1924.51.4.195

Schmidt, A. F. (2023). *The Socially Undesirable Sexual Selection Scale (SUSS)* [Manuscript in preparation]. Department of Psychology, Johannes Gutenberg University Mainz.

Spector, I. P., Carey, M. P., & Steinberg, L. (1996). The Sexual Desire Inventory: Development, factor structure, and evidence of reliability. *Journal of Sex & Marital Therapy*, *22*(3), 175–190. https://doi.org/10.1080/00926239608414655

Wiederman, M. W., & Allgeier, E. R. (1993). The measurement of sexual-esteem: Investigation of Snell and Papini’s (1989) Sexuality Scale. *Journal of Research in Personality*, *27*(1), 88–102. https://doi.org/10.1006/jrpe.1993.1006

1. Models were asked beforehand by phone whether they could put together a sexy outfit from their existing clothes and accessories; if they stated that they did not possess such attire or never went out in the evening, they were excluded from stimulus creation. [↑](#footnote-ref-1)
2. Apart from the two facial expression conditions relevant to the associated study (i.e., Flirting and Rejecting), all models also had photographs taken with a neutral facial expression in both clothing conditions. These are not further mentioned here for reasons of parsimony (for details, see Landwehr & Mundloch, 2023). [↑](#footnote-ref-2)
